# Promoting osteogenesis and bone regeneration employing icariin-loaded nanoplatforms

**DOI:** 10.1186/s13036-024-00425-4

**Published:** 2024-04-22

**Authors:** Mahsa Mohammadzadeh, Masoud Zarei, Hossein Abbasi, Thomas J. Webster, Nima Beheshtizadeh

**Affiliations:** 1 Department of Materials Engineering, Isfahan University of Technology, Isfahan, 84156-83111 Iran; 2 Department of Materials Science and Engineering, Sharif University of Technology, Tehran, Iran; 3 Department of Mechanical Engineering, University of Michigan-Dearborn, Dearborn, MI 48128 USA; 4 School of Health Sciences and Biomedical Engineering, Hebei University of Technology, Tianjin, China; 5 School of Engineering, Saveetha University, Chennai, India; 6 Program in Materials Science, UFPI, Teresina, Brazil; 7 Department of Tissue Engineering, Faculty of Advanced Medical Sciences, Tabriz University of Medical Sciences, Tabriz, Iran; 8 Regenerative Medicine Group (REMED), Universal Scientific Education and Research Network (USERN), Tehran, Iran

**Keywords:** Osteogenesis, Bone regeneration, Icariin, Nanoplatforms, Nanofibers, Nanoparticles

## Abstract

There is an increasing demand for innovative strategies that effectively promote osteogenesis and enhance bone regeneration. The critical process of bone regeneration involves the transformation of mesenchymal stromal cells into osteoblasts and the subsequent mineralization of the extracellular matrix, making up the complex mechanism of osteogenesis. Icariin’s diverse pharmacological properties, such as anti-inflammatory, anti-oxidant, and osteogenic effects, have attracted considerable attention in biomedical research. Icariin, known for its ability to stimulate bone formation, has been found to encourage the transformation of mesenchymal stromal cells into osteoblasts and improve the subsequent process of mineralization. Several studies have demonstrated the osteogenic effects of icariin, which can be attributed to its hormone-like function. It has been found to induce the expression of BMP-2 and BMP-4 mRNAs in osteoblasts and significantly upregulate Osx at low doses. Additionally, icariin promotes bone formation by stimulating the expression of pre-osteoblastic genes like Osx, RUNX2, and collagen type I. However, icariin needs to be effectively delivered to bone to perform such promising functions.

Encapsulating icariin within nanoplatforms holds significant promise for promoting osteogenesis and bone regeneration through a range of intricate biological effects. When encapsulated in nanofibers or nanoparticles, icariin exerts its effects directly at the cellular level. Recalling that inflammation is a critical factor influencing bone regeneration, icariin's anti-inflammatory effects can be harnessed and amplified when encapsulated in nanoplatforms. Also, while cell adhesion and cell migration are pivotal stages of tissue regeneration, icariin-loaded nanoplatforms contribute to these processes by providing a supportive matrix for cellular attachment and movement. This review comprehensively discusses icariin-loaded nanoplatforms used for bone regeneration and osteogenesis, further presenting where the field needs to go before icariin can be used clinically.

## Introduction

Bone disorders and fractures represent significant challenges in healthcare, particularly as the global population ages [1, 2]. These conditions not only cause debilitating pain and disability but also impose a substantial burden on healthcare systems worldwide. Understanding the background of bone disorders and fractures is crucial for appreciating the urgency and importance of advancements in bone regeneration, including the utilization of innovative approaches for providing a bone remedy [3]. Bone disorders encompass a wide range of conditions that affect the structure, strength, and function of bones [4].

Osteoporosis, characterized by reduced bone mineral density and increased bone fragility, is one of the most prevalent bone disorders, particularly among the elderly and postmenopausal women [5]. This condition significantly increases the risk of fractures, particularly in weight-bearing bones such as the hip, spine, and wrist. Osteoporotic fractures not only diminish the quality of life but also elevate mortality rates, posing a substantial public health concern [6]. Additionally, other bone disorders, such as osteogenesis imperfecta (OI) or brittle bone disease, are genetic disorders characterized by fragile bones prone to fracture even with minimal trauma [7–9]. These conditions primarily affect collagen production or quality, leading to bone deformities and recurrent fractures, often beginning in childhood. Traumatic fractures, resulting from accidents, falls, or sports injuries, are also prevalent, particularly among younger individuals [10]. These fractures can vary in severity, ranging from simple fractures that heal with proper immobilization to complex fractures requiring surgical intervention and prolonged rehabilitation.

Despite advancements in fracture management and surgical techniques, achieving optimal bone healing remains a challenge, especially in cases of severe trauma or compromised bone quality [11, 12]. Traditional approaches to fracture treatment, such as bone grafts and fixation devices, have limitations, including donor site morbidity, limited availability, and inadequate biomechanical properties [13]. Despite notable advancements in medical therapies, these challenges persist [14–16]. Consequently, there is an increasing demand for innovative strategies that effectively promote osteogenesis and enhance bone regeneration.

In response to these challenges, bone tissue engineering (BTE) has emerged as a promising approach to regenerate and repair damaged bone tissue [17, 18]. By integrating principles of biology, materials science, and engineering, BTE aims to develop biomimetic scaffolds, bioactive molecules, and cell-based therapies to promote bone healing and regeneration [19, 20]. BTE is an evolving field within regenerative medicine, aimed at addressing challenges posed by bone disorders, fractures, and critical-sized bone defects [21, 22]. The critical process of bone regeneration involves the transformation of mesenchymal stromal cells (MSCs) into osteoblasts and the subsequent mineralization of the extracellular matrix (ECM), making up the complex osteogenesis mechanism [23, 24].

Icariin (ICA), a natural flavonoid derived from plants of the epimedium genus, has a rich historical background in traditional Chinese medicine [25]. Commonly known as "horny goat weed", these plants have been used for centuries as remedies for various health conditions, including bone-related ailments [26, 27]. Its diverse pharmacological properties, such as anti-inflammatory, anti-oxidant, and osteogenic effects, have attracted considerable attention in biomedical research [28, 29].

On the other hand, regenerative medicine has significantly benefited from nanotechnology, which provides customized solutions for delivering drugs and engineering tissues [30]. Among the various nanoplatforms studied, nanofibers (NFs) and nanoparticles (NPs) have shown great potential for applications in BTE. NFs provide a three-dimensional (3D) scaffold mimicking the natural bone ECM, facilitating cell adhesion, proliferation, and differentiation [31, 32]. Also, NPs offer controlled drug release and enhanced bioavailability [33]. Exploiting the potential of icariin-loaded nanoplatforms presents an exciting avenue to promote osteogenesis and bone regeneration effectively. Numerous studies have explored the potential of icariin-loaded nanoplatforms in enhancing osteogenesis and bone regeneration, however, challenges such as bioavailability, regulations, and commercialization exist [29, 34, 35].

The main objective of this review is to thoroughly examine and analyze the various applications, mechanisms, and recent research advancements associated with ICA-loaded nanoplatforms in promoting osteogenesis and facilitating bone regeneration. Various techniques used for encapsulating ICA within NFs and NPs, discussing their biological and physical effects on BTE, are reviewed here. By collating and synthesizing the available literature, we intend to provide valuable insights into the current state-of-the-art in this field and identify potential avenues for future research and clinical translation.

## Icariin

### Structure and physicochemical properties

**Fig. 1:**
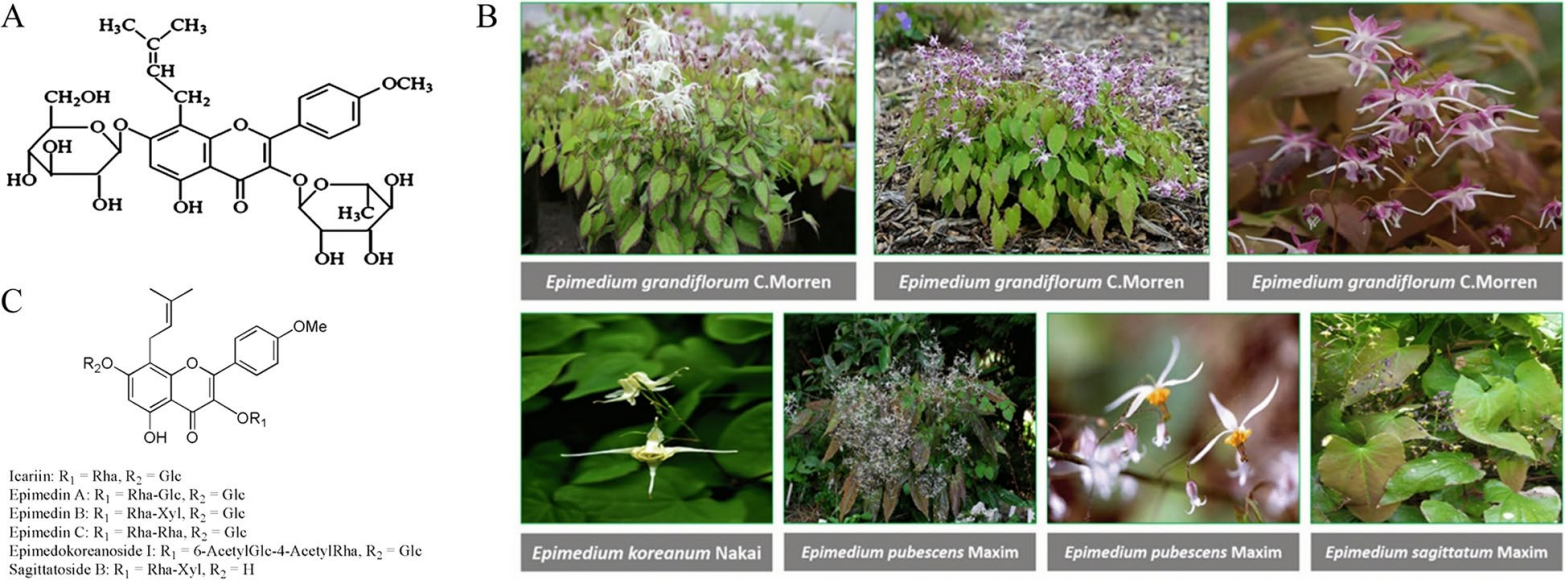
**A** The chemical structure of ICA (C_33_ H_40_ O_15_; molecular weight=676.67). Reprinted with permission from [41], **B** Various species of epimedium as sources of ICA and its derivatives. Reprinted with permission from [42], **C** Chemical structures of ICA derivatives. Reprinted with permission from [43]

Icariin, which is a prenylated flavanol glycoside, is a natural compound characterized by the chemical name 2-(4'-methoxylphenyl)-3-rhamnosido-5-hydroxyl-7-glucosido-8-(3'-methyl-2-butylen-yl)-4-chromanone [36, 37]. The chemical structure of ICA is characterized by its unique arrangement of functional groups, which contributes to its diverse pharmacological activities [38]. It consists of 33 carbon atoms, 40 hydrogen atoms, and 15 oxygen atoms. The molecular formula of ICA is C_33_H_40_O_15_, while its chemical structure is depicted in Fig. 1A. Its molecular weight is approximately 676.66 g/mol. The chemical structure of ICA features a flavone backbone, a common structural motif found in flavonoids [39]. At position 7 of the flavone core, ICA has a glucose (C_6_H_12_O_6_) moiety attached as a glycoside. Additionally, at position 8 of the flavone core, ICA is prenylated, meaning it has a prenyl group (C_5_H_8_) attached. This prenyl group is responsible for the compound's lipophilic nature and contributes to its bioactivity [40].

Moreover, ICA appears as a yellowish or pale yellow crystalline solid with a melting point ranging from 231 to 235 °C [44]. While it is sparingly soluble in water, it exhibits solubility in organic solvents such as ethanol and dimethyl sulfoxide (DMSO) [45]. This lipophilic nature plays a crucial role in facilitating its effective cellular uptake and interaction with biological membranes [46].

### Pharmacological properties

Icariin has been extensively studied for its diverse pharmacological properties, revealing its potential in various therapeutic applications. Firstly, it stimulates the formation and regeneration of bones by enhancing the differentiation and mineralization of MSCs, demonstrating remarkable osteogenic activity [47, 48]. Furthermore, ICA displays significant anti-inflammatory properties by effectively restraining the creation of pro-inflammatory cytokines and enzymes [49]. Its antioxidant potential provides cellular protection against oxidative stress-induced damage [50, 51].

ICA exhibits neuroprotective effects by promoting neuronal survival and mitigating neuroinflammation [52]. Also, it has been investigated for its cardiovascular benefits, showing promise in improving endothelial function and protecting against cardiovascular diseases [53, 54]. The multifaceted pharmacological activities of ICA make it an attractive candidate for various biomedical and therapeutic applications.

In addition to its diverse pharmacological activities, ICA has been found to mediate osteogenesis through its hormone-like function, as highlighted in studies [55, 56]. Notably, ICA has demonstrated the ability to induce the expression of bone morphogenetic protein-2 (BMP-2) and BMP-4 mRNA in osteoblasts. However, it should be highlighted that at a higher concentration of 10^-5^ M, ICA was toxic and hindered osteoblastic differentiation. Findings suggest that administering ICA after bone fracture can accelerate mineralization and osteogenesis, ultimately leading to significant improvements in bone healing. Accordingly, it has the potential to be an alternate therapy for postmenopausal osteoporosis and bone fractures.

### The source and derivatives

Icariin is a prenylated flavanol glycoside, meaning it consists of a flavanol structure with a prenyl (3-methyl-2-butenyl) group attached to it. ICA, which is obtained from different epimedium plant species like epimedium brevicornum maxim., epimedium pubescens maxim., and epimedium sagittatum (Sieb. et Zucc.) maxim, is extracted for its medicinal benefits, as shown in Fig. 1B [25, 42, 57].

In addition to ICA, the predominant active compound in epimedium species, there exists a range of derivatives and related compounds that have garnered attention for their potential biological activities [58]. Notable among these derivatives is icariside I, which emerges as a hydrolysis product of ICA resulting from the cleavage of the glycosidic bond connecting the glucose and flavanol components. Icariside I retains some of the bioactivities that are attributed to ICA.

Another significant derivative, desmethylicaritin, shares structural similarities with icariin but lacks the glucose molecule due to hydrolysis. This derivative exhibits estrogenic effects. Prenylated flavanol glycosides, identified as epimedins A, B, and C, are present in various epimedium species. These derivatives bear structural resemblance to ICA and could contribute to the overall bioactivity of these plants [43].

Another isolated derivative, epimedokoreanin A, hailing from epimedium koreanum, demonstrates anti-inflammatory and antioxidant properties. The exploration of these derivatives expands our understanding of the broader pharmacological potential inherent in epimedium species beyond the primary ICA compound [59–61]. In Fig. 1C, the common chemical structures of ICA derivatives are presented.

It's important to note that the specific species of epimedium, growing conditions, and extraction methods can influence the composition of derivatives present in plant extracts. Researchers often study these derivatives to understand their potential health benefits and explore their various pharmacological activities, including osteogenesis promotion [62], and anti-inflammatory effects [63]. As with any natural compound, the pharmacological effects of ICA and its derivatives are subject to ongoing research, and their mechanisms of action and potential applications continue to be explored in both traditional medicine and recent studies.

Previous studies reported that icariin was extracted from the aerial parts of plant Epimedium koreanum (Berberidaceae) with hot water and purified by hydrophobic interaction chromatography, ion-exchange chromatography and finally by crystallization [64]. A previously reported dual-mode high-speed counter-current chromatographic technique has been devised for the efficient extraction and purification of icariin derived from Epimedium. Li et al. [65] claimed that the unrefined icariin was acquired by the process of extracting with ethyl acetate and ethanol from the dried above-ground sections of Epimedium using sonication. Upon their report, a dual-mode approach was used to execute high-speed counter-current chromatography using a two-phase solvent system consisting of n-butanol-ethyl acetate-water (3:7:10, v/v) [65]. Also, Du et al. [66] reported that icariin was purified from the extract of Epimedium segittatum by high-speed counter-current chromatography with a two-phase solvent system composed of n-hexane-n-butanol-methanol-water (1:4:2:6, v/v). Accordingly, the extraction and purification procedures of icariin is considered as a well-known process.

### Mechanism and bioavailability

Understanding the mechanisms of action and factors affecting the bioavailability of ICA and its derivatives is crucial for optimizing their therapeutic potential. Researchers continue to investigate novel delivery strategies and formulations to improve the bioavailability of these compounds and enhance their effectiveness in various therapeutic applications.

### Mechanism of action

Icariin, as a multifunctional flavonoid, operates through a diverse mechanism of action, yielding a spectrum of advantageous effects that collectively make it a potential candidate for various therapeutic applications. It plays a prominent role in promoting osteogenesis by activating key signaling pathways that are responsible for bone formation [67]. The expression of osteogenic markers, such as osteopontin, osteocalcin, and collagen type I, is also improved through its use [39, 40]. In addition, ICA enhances the production of bone morphogenetic proteins (BMPs), like BMP-2 and BMP-4, which are vital for guiding osteoblast differentiation and mineralization [48, 68].

At lower doses, ICA significantly upregulates the osteogenic-specific transcription factor, Osterix (Osx) [69, 70]. Furthermore, it enhances the expression of pro-osteoblastic genes like Osx, RUNX2, ALP, and collagen type I, thereby promoting bone formation. ICA regulates the osteoprotegerin/receptor activator of nuclear factor-κB ligand (OPG/RANKL) signaling pathway in osteoclasts, resulting in inhibitory effects on bone resorption [71].

Additionally, ICA has been shown to impede the formation of fat cells in bone marrow-derived mesenchymal stromal cells (BMSCs) and boost their dedication to becoming bone cells [72]. According to Fan et al.'s research [73], ICA was found to increase the growth of BMSCs in a dose-dependent manner. Additionally, it was discovered that even at very low concentrations between 10^-9^ M and 10^-6^ M, ICA promoted osteoblastic differentiation.

In addition to its effects on bone health, ICA demonstrates potent anti-inflammatory properties by effectively suppressing the production of pro-inflammatory cytokines, including interleukin-6 (IL-6) and tumor necrosis factor-alpha (TNF-α) [74]. This control is enacted through the modulation of nuclear factor-kappa B (NF-κB), a central regulator of inflammation, ultimately proposing ICA as a candidate for managing chronic inflammatory conditions. Its antioxidant prowess shines in scavenging free radicals and inhibiting oxidative stress-induced damage, concurrently bolstering the expression of essential antioxidant enzymes like superoxide dismutase (SOD) and catalase (CAT) [75].

These attributes collectively protect cellular integrity and suggest ICA's potential in addressing oxidative stress-related disorders. Moreover, ICA's neuroprotective effects manifest through the promotion of neuronal survival and the inhibition of neuroinflammation. Its modulation of neurotransmitter levels and reduction of oxidative stress underscore its potential role in mitigating neurodegenerative disorders [52]. This multifaceted mechanism underscores ICA's potential in diverse therapeutic arenas, from BTE to neuroprotection and inflammation management.

#### Bioavailability

Bioavailability encompasses the fraction of a compound that enters systemic circulation, thereby becoming accessible for biological activity [76]. The biological activities of ICA are attributed to its interactions with multiple molecular targets and signaling pathways [40]. Several important pathways, such as the ones involving BMPs, Wnt/β-catenin signaling, and RUNX2, contribute to both osteogenic differentiation and bone formation processes. These pathways are crucial in regulating osteogenic differentiation and promoting bone formation [77, 78]. Numerous factors influence the bioavailability of ICA and its derivatives, thereby impacting their therapeutic potential.

First, the limited solubility of ICA in water restricts its absorption within the gastrointestinal tract, motivating the exploration of formulations to enhance solubility, including approaches like NPs or inclusion complexes, to amplify bioavailability. Second, metabolic conversions of ICA within the liver and intestine yield diverse metabolites, with structural variations that can influence their bioactivity and pharmacokinetics [76].

Third, oral administration of ICA subjects it to the first-pass effect, a process wherein liver metabolism precedes systemic circulation, potentially diminishing bioavailability. Fourth, diverse delivery systems such as NPs, liposomes, and solid lipid NPs have been investigated to augment bioavailability. These systems enhance solubility, shield ICA from degradation, and facilitate absorption. Lastly, the pharmacokinetic profiles of certain ICA derivatives might deviate from the parent compound, introducing changes in metabolism, absorption, and distribution that affect bioavailability [45, 79].

Recent studies showed that to improve the bioavailability of ICA, it is used in combination with other materials, including polymers and hydrogels [45, 80]. A study by He et al. [81] utilized hydrogels made from hyaluronic acid and icariin (HA-ICA). Also, they created a hydrogel by combining methacrylic anhydride-icariin (MA-ICA) and methacrylic anhydride-hyaluronic acid (HAMA) through photopolymerization. The HA-ICA hydrogel successfully provided sustained bioactivity by releasing icariin in a controlled manner over a prolonged time.

Yu et al. [82] explored an innovative approach to enhance immunogenic cell death in chemotherapy-resistant hepatocellular carcinoma (HCC) using ICA. They discovered that combining ICA and doxorubicin effectively triggered potent immunogenic cell death in both mouse Hepa1–6 and human Huh7 HCC cells. This outcome was accomplished by activating mitophagy and apoptosis pathways. Additionally, the use of polymeric NPs for targeted delivery of ICA and doxorubicin resulted in a remodeling of the immunosuppressive tumor microenvironment. As a result, a robust immune memory response was elicited, leading to a significant improvement in the early-stage anti-HCC efficacy in a mouse model [82]. However, the clinical translation of ICA is hindered by its limited bioavailability, resulting from its hydrophobic nature and rapid enzymatic metabolism. To address this challenge, researchers have turned to nano-based delivery systems, such as NFs and NPs, to encapsulate ICA and improve its therapeutic potential.

## Icariin-loaded nanoplatforms

### Nanofibers

Nanofibers have captured significant attention in recent times due to their distinct characteristics and versatile applications across various domains like biomedicine, electronics, and environmental engineering [83]. Electrospinning is a technique widely used for creating NFs due to its cost-effectiveness and flexibility. It enables precise control over the structure and composition of the fibers by utilizing electric fields to extract polymer solutions or melts into extremely fine fibers, generally on a nanoscale [84, 85]. In the realm of therapeutic applications, electrospun NFs loaded with bioactive compounds have exhibited notable potential.

#### Electrospinning

Electrospinning, a versatile technique for fabricating nanofibers, has diverse applications, including pharmaceuticals [86, 87]. In the case of ICA, a natural compound with potential health benefits, various electrospinning methods can be employed to craft nanofibers for controlled release and delivery. For instance, Gong et al. [88] developed an electrospun fibrous membrane enclosing ICA within PCL/gelatin NFs to create an artificial periosteum for bone grafts. The incorporation of ICA enhanced hydrophilicity, pre-osteoblast proliferation, and osteogenic differentiation, making the developed membrane a promising candidate for promoting bone regeneration.

**Fig 2:**
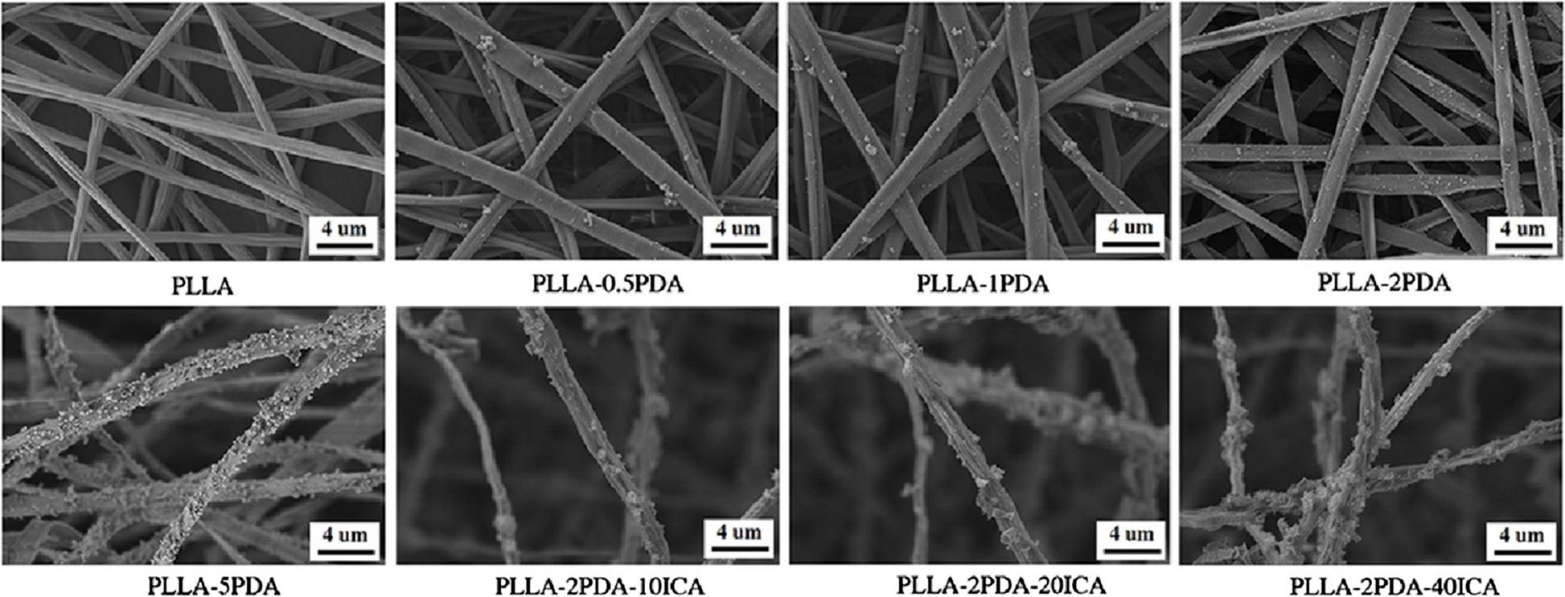
Morphological enhancement of fibrous membranes for BTE. PLLA: poly(l-lactide), PDA: polydopamine, ICA: Icariin. Reprinted with permission from [89]

Liu et al. [89] conducted a study where they developed a new technique to improve the compatibility and bone-forming activity of electrospinning poly(l-lactide) (PLLA) fibrous membranes. They achieved this by applying a polydopamine (PDA) adhesive coating to immobilize ICA (Fig. 2). The study found that the PLLA-2PDA-ICA fibrous membranes, with different concentrations of ICA, resulted in enhanced cell adhesion, proliferation, and differentiation into bone cells (MC3T3-E1). These findings suggest that their developed fibrous membranes have potential applications in BTE [89].

**Fig. 3:**
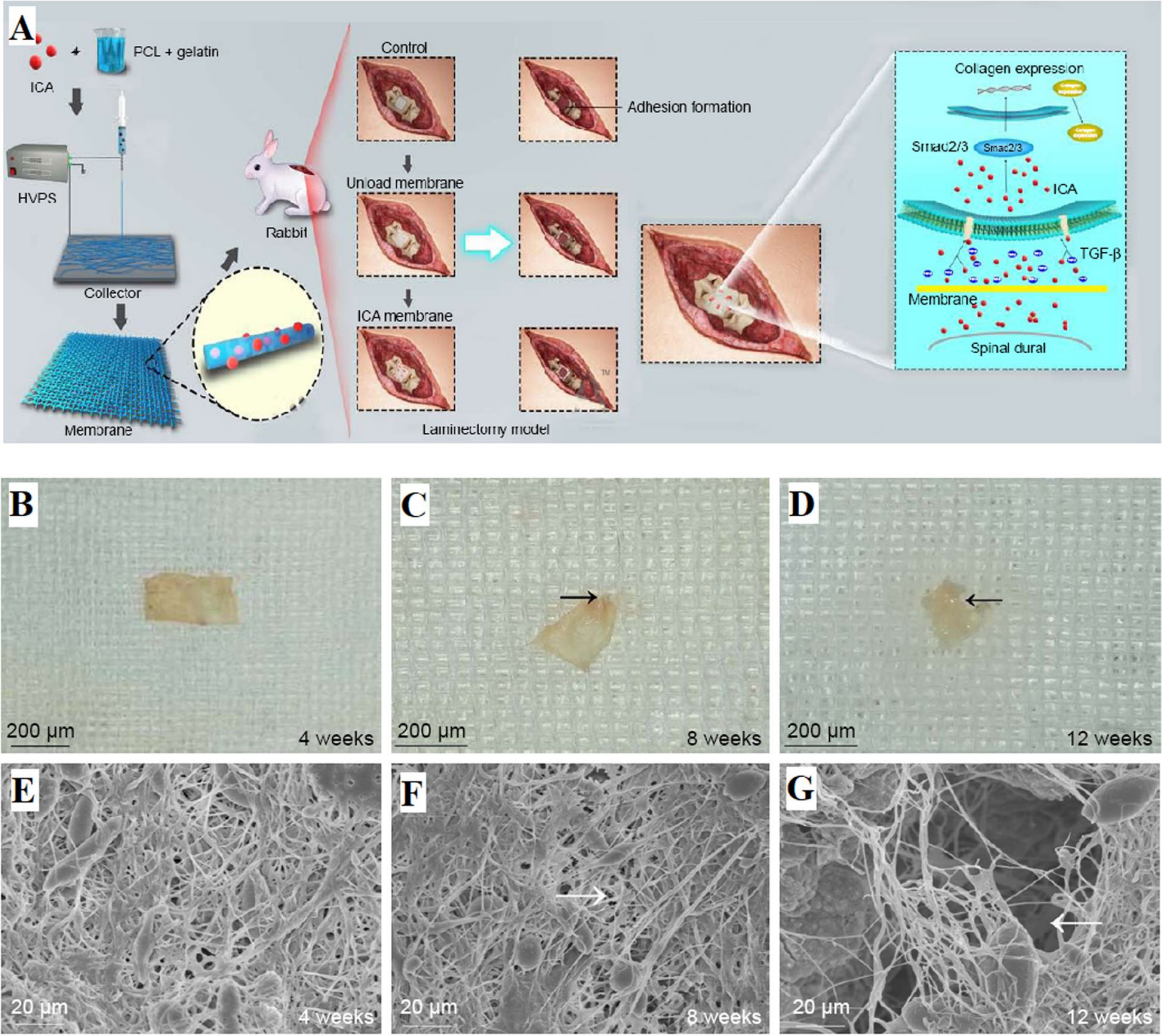
**A** Diagram depicting the creation of the PCL-gelatin membrane and its application in a laminectomy model for adhesion prevention. Additionally, a representation highlighting potential modes of inhibiting TGF-β and Smad pathways using ICA, **B-D** Morphological characterization of ICA-loaded PCL-gelatin membrane, **E–G** SEM images of membrane surfaces captured at various post-implantation time points. ICA: Icariin, HVPS: high voltage power supply. The black arrows indicate the absorbed margin, and the white arrows indicate the pores. Reprinted with permission from [90]

Huang et al. [90] developed a new sub-microfiber mat made of electrospun PCL and gelatin, loaded with ICA, to prevent adhesions in the epidural space after laminectomy. The ICA-loaded membranes effectively controlled and released ICA, inhibiting fibroblast adhesion and proliferation in laboratory tests and reducing epidural adhesion in rabbit laminectomy models. This was achieved by regulating the TGF-β and Smad pathways (Fig. 3A). These ICA-loaded electrospun membranes made of PCL and gelatin have significant potential as nanomaterials to prevent adhesion formation after laminectomy in clinical applications (Fig. 3B-G). However, further clinical trials are necessary to evaluate their safety for future clinical use.

**Fig. 4:**
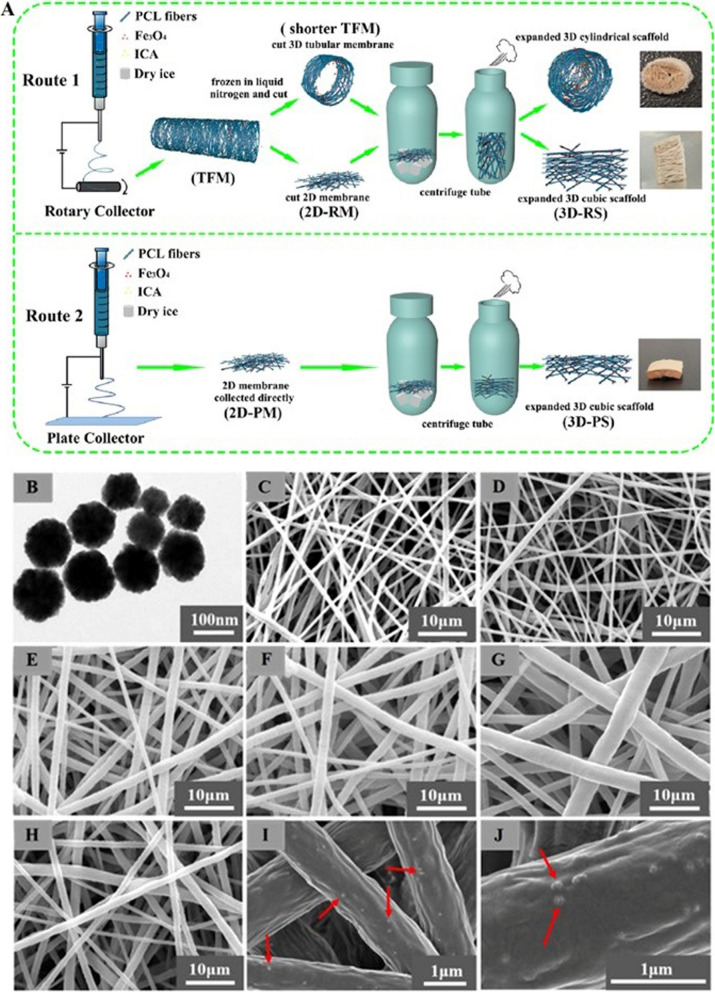
**A** The production processes of the PCL/Fe_3_O_4_/ICA 2D membrane and 3D scaffold. The tubular fibrous membrane obtained from the rotary device is denoted as TFM, while the 2D membrane directly collected from the flat plate is labeled as 2D-PM. Additionally, 2D-RM represents the 2D membrane cut from TFM collected using the rotary device. The scaffold derived from 2D-PM is termed 3D-PS, and the scaffold originating from 2D-RM is named 3D-RS. **B-J** The transmission electron microscopy (TEM) image shows Fe_3_O_4_ MNPs, while the scanning electron microscopy (SEM) images showcase various compositions of 2D-RMs collected by the rotary collector. These compositions include: **B** Fe_3_O_4_ MNPs; **C** PCL; **D** PCL/ICA; **E** PCL/Fe_3_O_4_-0.25%; **F** PCL/Fe_3_O_4_-0.5%; **G** PCL/Fe_3_O_4_-1%; and **H-J** PCL/Fe_3_O_4_/ICA. The highlighted rows indicate the presence of Fe_3_O_4_ MNPs. Reprinted with permission from [91]

Li et al. developed a novel electrospinning method, as shown in Fig. 4, to fabricate a 3D magnetic fibrous scaffold containing ICA and Fe_3_O_4_ magnetic nanoparticles (MNPs). The scaffold, as indicated in Fig.4, exhibited enhanced cell viability, infiltration, collagen deposition, and angiogenesis, demonstrating its promising potential for BTE applications, especially under the influence of a static magnetic field (SMF). The transmission electron microscopy (TEM) image of Fe_3_O_4_ MNPs images with different compositions collected by rotary collector are provided in Fig. 4B [91].

**Fig. 5:**
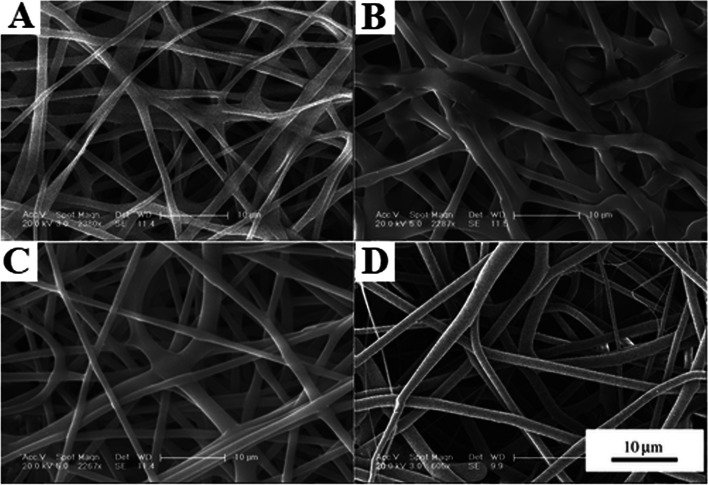
SEM images of the fibrous scaffolds: **A** PLGA fibrous scaffold; **B** PLGA/0.01% ICA fibrous scaffold, **C** PLGA/0.1% ICA fibrous scaffold, and **D** PLGA/1% ICA fibrous scaffold. The scale bar corresponds to 10 µm. Reprinted with permission from [92]

Zhao et al. [92] explored a novel electrospinning method to create a composite scaffold of poly(lactide-co-glycolide) (PLGA) loaded with icariin (Fig. 5). Demonstrating the slow and gradual release of ICA from the scaffold suggests its potential for inhibiting osteoarthritis progression. The scaffold displayed remarkable biocompatibility and encouraged the production of the ECM, preserving the functional structure of articular cartilage and preventing the resorption of subchondral bone trabeculae. These findings indicate the therapeutic potential of the scaffold in treating osteoarthritis [92].

Liu et al. [93] recently developed a new technique to create a dual fibrous PLLA/chitosan (CS) membrane with a hierarchical structure. This membrane was then treated with ICA and deferoxamine (DFO) using a PDA coating. The resulting PLLA/CS-PDA/ICA/DFO membrane exhibited improved compatibility with cells, as well as enhanced bone formation and blood vessel growth, surpassing the effectiveness of membranes with only one or neither ICA nor DFO immobilization. These findings suggest that the PLLA/CS-PDA/ICA/DFO membrane has great potential as a substitute for bone repair, as it simultaneously enhances osteogenesis and angiogenesis [93].

#### Core-shell electrospinning

Core-shell electrospinning is an advanced technique used to produce nanofibers with a distinct core and shell structure. In the context of ICA usage, this method involves the creation of core-shell NFs with ICA encapsulated in the core and a polymer shell surrounding it. The process utilizes a coaxial spinneret, through which two different solutions are simultaneously electrospun. The inner core solution contains ICA, while the outer shell solution consists of a polymer material.

The sheath-fluid coaxial flow in the electrospinning process leads to the formation of core-shell NFs. Rapid solidification of the outer polymer shell occurs due to the electric field applied between the spinneret and the collector, trapping the ICA-loaded core solution inside. This configuration enables controlled and sustained release of ICA from the nanofibers.

**Fig. 6:**
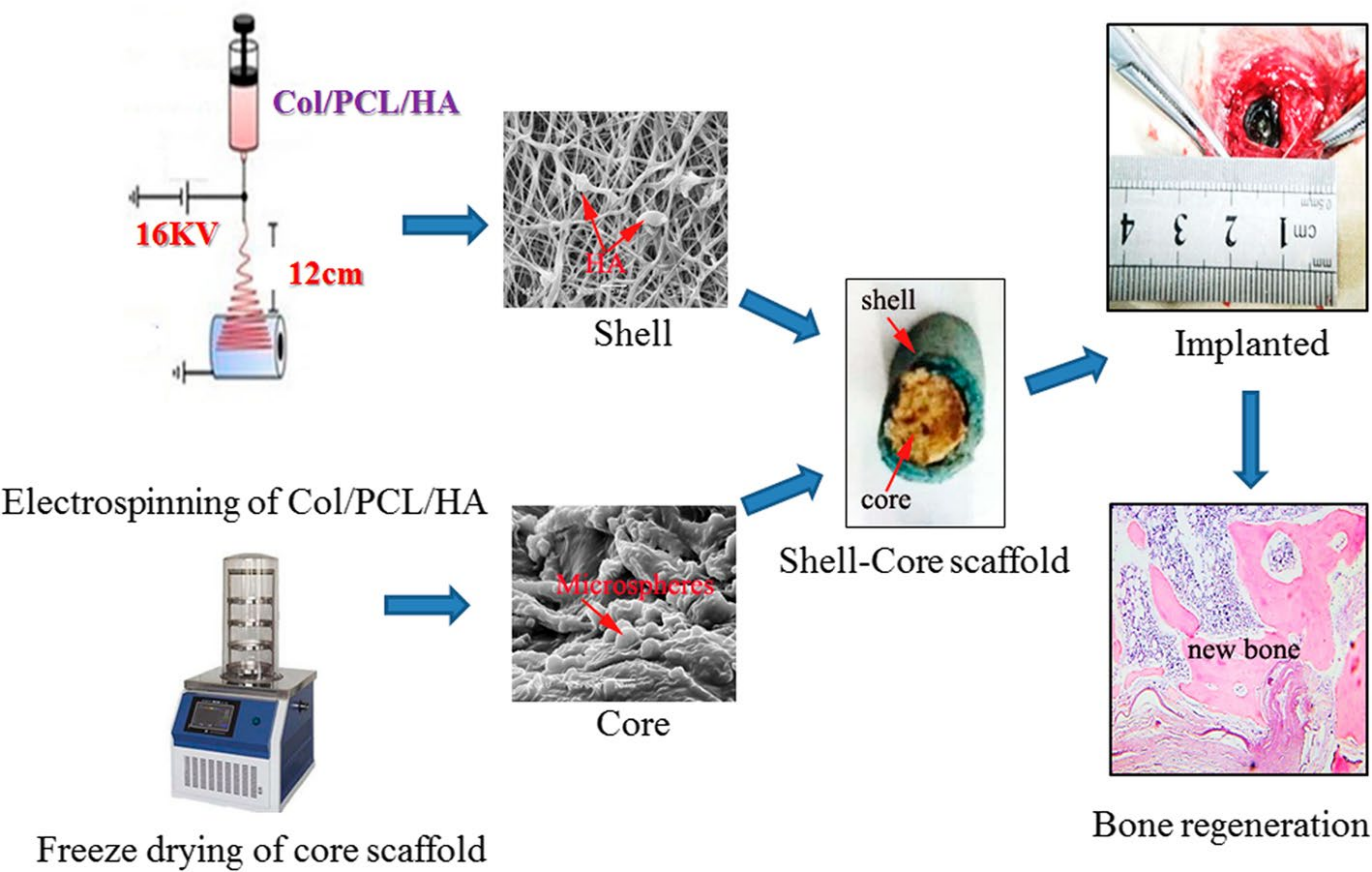
Schematic representation outlining the process involved in creating a composite scaffold of collagen/PCL/hydroxyapatite/ICA. Col: Collagen, PCL: Polycaprolactone, HA: hydroxyapatite, Reprinted with permission from [94]

Overall, core-shell electrospinning is a promising technique for the production of NFs tailored for ICA usage. Its ability to provide controlled release, improved stability, and potential for targeted delivery makes it a valuable approach for developing nanofiber-based systems for ICA delivery in various pharmaceutical applications. In a study conducted by Zhao et al. [94], they achieved the successful fabrication of innovative drug-loading core-shell composite scaffolds made from collagen, polycaprolactone (PCL), and hydroxyapatite. This was accomplished through a combination of electrospinning and freeze-drying techniques. The incorporation of ICA into the core scaffolds was a significant aspect of their work (Fig. 6). They reported that the developed ICA-loaded scaffolds exhibited a superior potential for bone regeneration, surpassing the scaffolds without ICA in their ability to stimulate bone regrowth. This is due to the combined effects of ICA release and hydroxyapatite, making them highly promising for BTE and repairing bone defects [94].

Moreover, Xu et al. [95] successfully synthesized poly(N-isopropylacrylamide) (PNIPAAm)-polyethylene glycol (PEG) micelles with a core-shell morphology using reversible addition fragmentation chain transfer (RAFT) polymerization. These micelles exhibited temperature-sensitive characteristics, with a lower critical solution temperature (LCST) between 34~42°C. The PNIPAAm: PEG ratio of 1.8:1 resulted in an LCST of 37.8°C, which is similar to body temperature. This suggests that ICA could be effectively delivered using this ratio as a drug carrier. The micelles demonstrated a high ICA encapsulation efficiency of approximately 85%, and the release of ICA was dependent on temperature. This makes icariin-PNIPAAm-PEG a promising option for treating bone defects and diseases, especially in the field of BTE.

Abdullah et al. [96] reviewed the design, roles, and controllable release strategies of core-shell fibers in tissue engineering and drug delivery. They discussed the advantages of core-shell fibers in preserving the bioactivity of sensitive biomolecules and achieving therapeutic effects through controlled release. Their work highlighted the importance of core-shell structure in tissue regeneration and drug delivery applications, as well as the challenges and future directions in this field [96]. In another study, the researchers managed to successfully create drug-loaded microspheres with a core-shell structure using silk fibroin and PLGA. These microspheres contained the pro-angiogenic drug tetramethylpyrazine (TMPZ) in the shell layer and the pro-osteogenic drug ICA in the core layer. This setup allows for the sequential release of the drugs, which promotes early angiogenesis and late osteogenesis at the specific site of bone defects. This innovative drug delivery system, with its dual-step release mechanism, showed great potential for clinical applications in the treatment of bone defects [97].

#### Coaxial electrospinning

Coaxial electrospinning is a modified technique where two solutions are simultaneously electrospun through a coaxial spinneret. In this method, a core fluid containing icariin is surrounded by a polymer shell solution. As the coaxial jet is ejected and travels to the collector, the solvents evaporate, leading to the formation of core-shell nanofibers. Coaxial electrospinning enables the encapsulation of ICA within the core, protected by the polymer shell, offering enhanced stability and controlled release properties.

Liu et al. [98] implemented a novel method to create a 3D scaffold with ICA for bone regeneration. This approach involved the use of coaxial electrospinning and electrospraying techniques. The scaffolds loaded with the drug displayed strong mechanical properties and stability. The controlled release of ICA within the scaffold facilitated increased ALP activity and deposition of ECM minerals in the MSCs. Consequently, this created a beneficial environment for osteogenic applications in BTE [98].

They also harnessed the power of coaxial electrospinning in their research on developing a 3D MSCs-laden scaffold for bone regeneration [98]. By encapsulating ICA within zein/PLGA nanofibers, they created a scaffold with excellent mechanical properties and stability. The sustained release of ICA from this scaffold significantly enhanced the activity of ALP and the deposition of ECM minerals, ultimately promoting rapid bone regeneration. The study not only demonstrated the biocompatibility of the nanofiber scaffolds but also underscored their potential in BTE. These scaffolds provided an osteogenic environment that encourages MSCs to contribute to bone formation [98].

**Fig 7:**
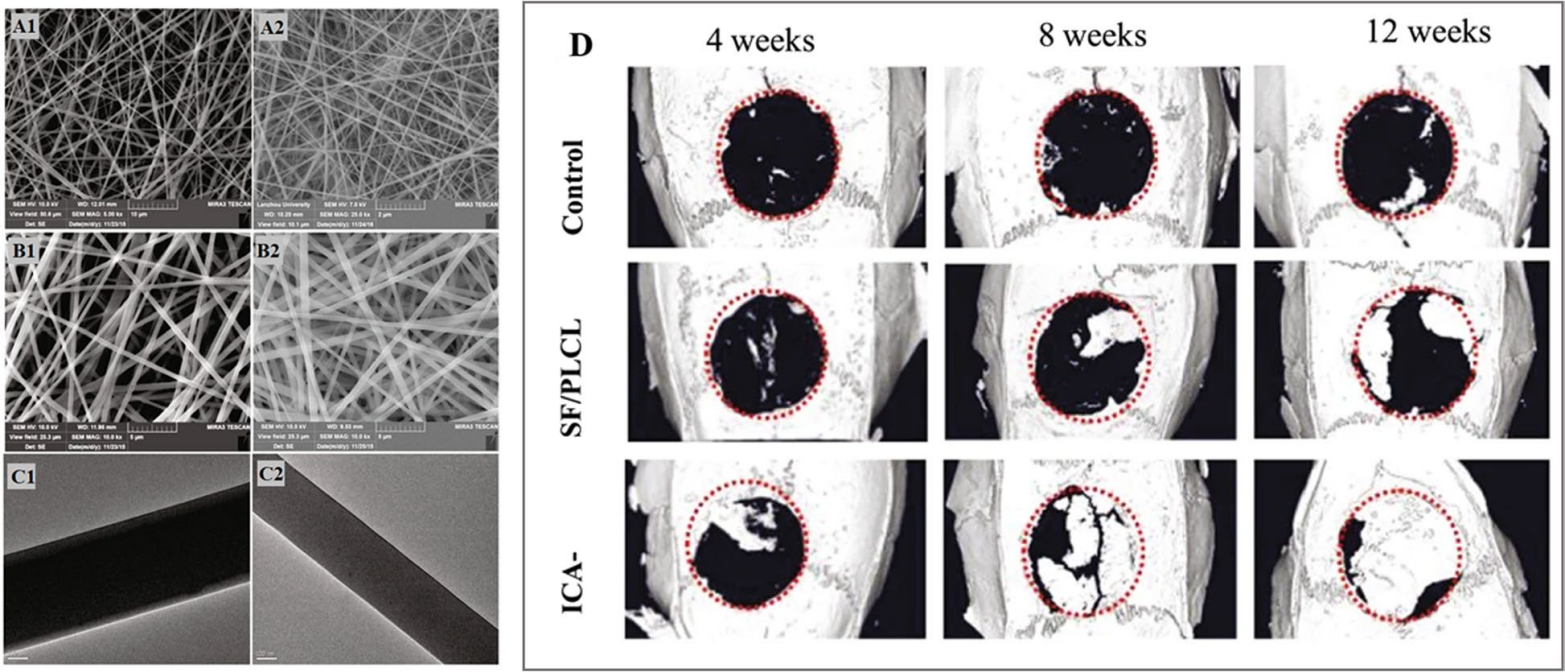
**A** SEM micrographs of the ICA-SF/PLCL nanofibrous membrane; **B** SEM micrographs of the SF/PLCL nanofibrous membrane; **C** TEM micrographs of the mentioned nanofibrous membrane (scale bars: A1: 10 μm; A2: 2 μm; B1 and B2: 5 μm; C1 and C2: 100 nm); **D)** μ-CT images of calvaria defects. Reprinted with permission from [99]

Yin et al. [99] pioneered the development of a novel silk fibroin (SF)/poly(l-lactide-co-caprolactone) (PLCL) nanofibrous membrane using coaxial electrospinning, with the aim of incorporating ICA (Fig. 7A-C). This membrane demonstrated the sustained and controlled release of ICA, resulting in excellent biocompatibility and enhanced osteogenic activity of BMSCs in vitro. Impressively, in vivo experiments using rat craniums exhibited significant bone repair after 12 weeks of implantation as shown in Fig. 7D. This study highlighted the potential of the ICA-loaded SF/PLCL nanofibrous membrane as a biomaterial for guided bone regeneration (GBR) in dental implants. The coaxial electrospinning technique proved to be effective in fabricating osteoinductive GBR membranes, offering a sustained delivery system for ICA that promotes bone regeneration without cytotoxicity [99].

#### Emulsion electrospinning

Emulsion electrospinning involves the dispersion of ICA in an emulsion system, where the active compound is dissolved or dispersed in an immiscible liquid phase. The emulsion is then electrospun, resulting in the formation of NFs with ICA incorporated within the polymer matrix. This method offers advantages such as improved solubility, controlled release, and enhanced bioavailability of ICA.

In this regard, Elyaderani et al. [100] conducted a comprehensive review on the techniques used for encapsulating ICA in NFs, with a specific focus on recent work involving hydroxyapatite-loaded scaffolds with core/shell nanofibers utilizing emulsion electrospinning. The study emphasized the significant advancements in scaffolds for hard tissue regeneration facilitated by emulsion electrospinning. It underscored the potential of this technique for constructing complex architectures capable of incorporating various bioactive agents, including ICA. However, challenges such as precise drug placement were also acknowledged. The study emphasized the necessity for in vivo validation of these systems before their application in clinical trials and commercial medical devices [100], as in vivo evaluation is considered as a pre-clinical evaluation and needed for further clinical trials [101].

#### Electrospun blend nanofibers

**Fig. 8:**
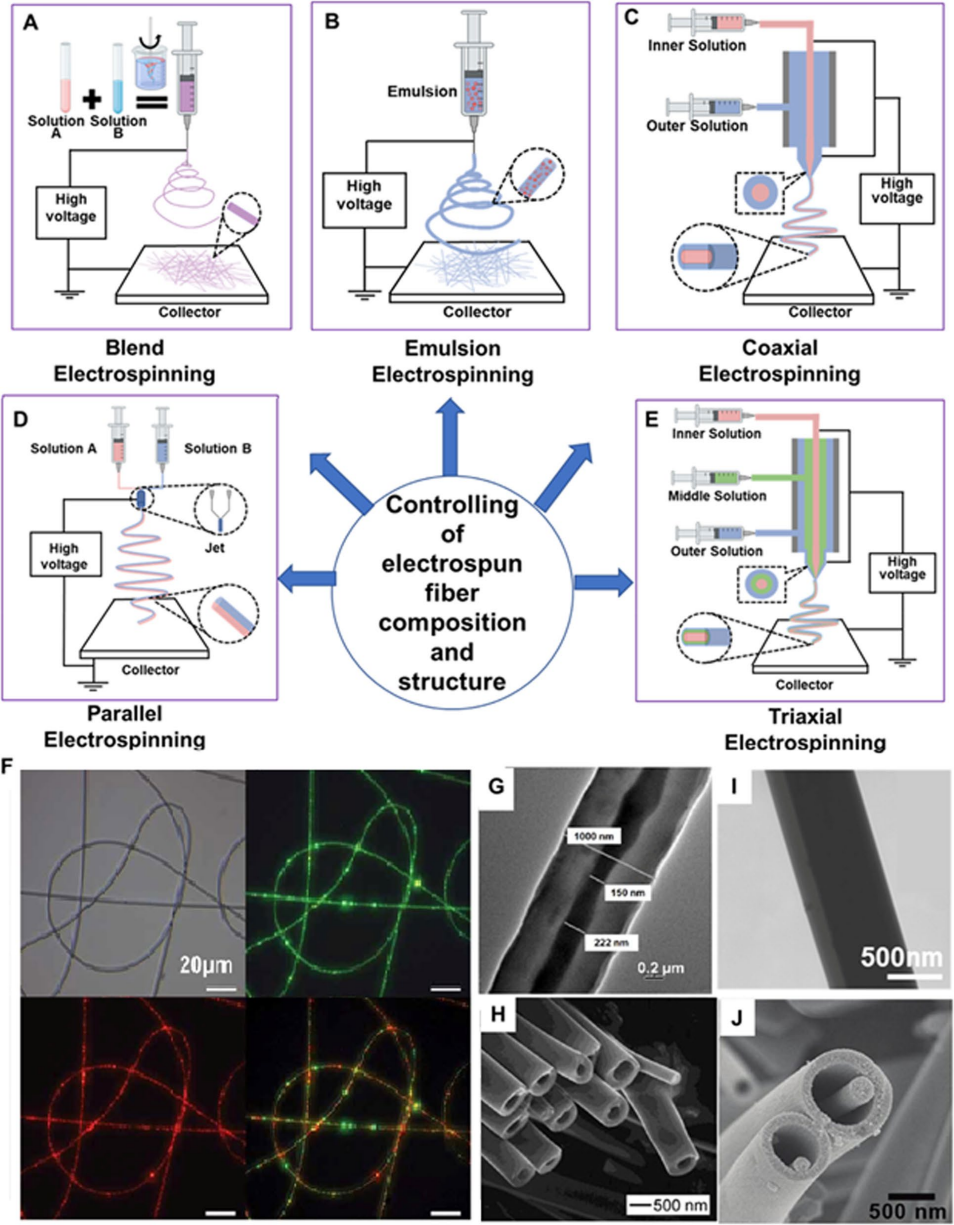
Innovative electrospinning techniques for controlling fiber composition and structure. This figure presents a schematic representation of: **A** blend electrospinning; **B** emulsion electrospinning; **C** coaxial electrospinning; **D** parallel electrospinning; and **E** triaxial electrospinning. **F** Emulsion electrospinning is further showcased through images of fabricated fibers, including optical and fluorescence images of polyurethane electrospun fibers containing PVA/EGF-AF488 and PVA/BSA-TR particles. The core-sheath structure achieved by coaxial electrospinning is displayed in a **G** TEM image; while **H** hollow fibers produced by combining coaxial electrospinning with core layer removal are depicted in an SEM image. Furthermore, **I** a Janus structural fiber from parallel electrospinning is shown in a TEM image; and **J** a fiber in tube structure resulting from triaxial electrospinning and middle layer removal is exhibited in an SEM image. All images are reprinted with permission from [102].

In this method, ICA can be incorporated into a polymer blend solution, where two or more polymers are mixed with the active compound. The blend solution is electrospun, and the resulting nanofibers contain a combination of polymers with ICA dispersed within. This approach allows for tailoring the properties of the nanofibers, such as mechanical strength, drug release kinetics, and compatibility with specific applications. These various electrospinning methods are shown in Fig. 8.

Offering flexibility in designing nanofibers for ICA usage, enables the controlled release, protection, and targeted delivery of the active compound for various pharmaceutical applications. Coaxial electrospinning is regarded as a more advantageous method for drug encapsulation compared to blend or emulsion electrospinning techniques. This is mainly because coaxial electrospinning has the ability to load the drug into the inner layer of fibers. This results in a significant decrease in the initial burst release of the drug and allows for a prolonged and controlled drug release [102–104].

As exemplified in the referenced studies, a comparative analysis reveals that several electrospinning techniques and material compositions have been explored in various research [94, 105]. Core-shell electrospinning has emerged as a promising technique, offering controlled release and improved stability of ICA within NFs. This approach encapsulates ICA within the core, as demonstrated by various studies. Also, coaxial electrospinning was utilized as one of the most promising strategies for developing ICA-loaded NFs, which offer precise control over ICA release and enhanced stability of NFs. A substantial fact is to note that composite scaffolds, combining ICA with other biomaterials like hydroxyapatite, showed great potential for stimulating bone regrowth and repairing defects.

### Nanoparticles

ICA's low bioavailability remains one of its major limitations [106]. This poor absorption and low oral bioavailability (~12%), due to the low membrane permeability and water solubility as well as a relatively low dissolution rate in biological solutions, has resulted in their limited clinical applications [107]. As a result, a delivery vehicle/system is urgently needed to enhance the bioavailability and sustained release of ICA. A number of particle-based approaches have been taken in order to address this issue elaborated hereunder.

#### Micelles

Polymeric micelles are nanoscale structures that self-assemble, consisting of amphiphilic polymers. These micelles possess a core-shell architecture, with the hydrophobic inner core capable of encapsulating hydrophobic drugs. The hydrophilic outer shell of the polymeric micelles, on the other hand, accommodates hydrophilic bioactive molecules. Between the core and the shell, an intermediate region with polar characteristics exists [108]. Above a certain concentration in aqueous solutions, micelles are formed through a critical micelle concentration or critical aggregation concentration [109]. Several methods are used for preparing micelles, including solvent casting, direct dissolution, dialysis, exfoliation-adsorption, nanoprecipitation, template synthesis, and interfacial polymerization [110, 111]. Three varieties of polymers are frequently employed in micelle fabrication: diblock copolymers, which consist of polystyrene and PEG [112]; triblock copolymers, which encompass polyethylene oxide (PEO) [113]; and graft copolymers, which include stearic acid and chitosan [114]. Multiple advantages in using polymeric micelles for drug delivery could be considered, such as their target specificity, tissue penetration ability, high drug-loading capacity, high stability, biodegradability, biocompatibility, low toxicity, and controlled-release features [115, 116].

In recent studies, ICA-loaded micelles have been found to be effective in improving ICA stability, solubility, and bioavailability for various diseases. For the purpose of enhancing pharmacokinetics and prolonging blood circulation time, an innovative polymeric micelle, composed of a self-assembled micelle between poly (D-lactic acid)-poly(N-isopropyl acrylamide) (PDLA-PNIPAM) and PEG-PLLA was developed to encapsulate ICA [117]. This polymeric micelle formulation of ICA demonstrated excellent stability and uniform dispersion over 48 hours. Additionally, this novel formulation was demonstrated to provide a sustained release of ICA both in vitro and in vivo.

Another study combined the hydrophobic section of ICA as the core with the hydrophilic section of the carboxylated mPEG micelle shell in an aqueous solution through esterification [118]. This resulted in an increase in icariin release from mPEG-ICA NPs in moderately acidic solutions, but hardly in neutral solutions, which has medical significance for the treatment of ischemic cardiomyopathy.

#### Structural transformation

Solid materials are frequently characterized by isostructurality and polymorphism, which have profound implications in nutritional supplements and medicine. The physicochemical properties of solid bioactive agents may vary depending on their polymorphs, including dissolution behavior, stability, and fusion enthalpy, thereby affecting their bioavailability [119]. Additionally, polymorphism pertains to the existence of multiple crystalline structures exhibited by a chemical substance, whereas isostructurality refers to the arrangement of packing within a chemical substance. Lina et al. [120] reported the synthesis of two pure forms of ICA polymorphs along with ten solvate modifications, including α anhydrous polymorphs. Among these forms, the α anhydrous polymorph demonstrated enhanced characteristics compared to ICA, including reduced hygroscopicity, increased water solubility, and improved thermal stability.

#### Extracellular vesicles

Extracellular vesicles (EVs) are nanoscale membranous particles derived from various living cells, which have gained significant attention recently for their various therapeutic uses, specifically their efficiency in delivering medications [121]. The capacity of electric vehicles to encapsulate drugs that are both hydrophobic and hydrophilic is a major benefit [122]. Zhang et al. [123] carried out a study where they loaded ICA into bovine milk EVs using a co-incubation method. The drug loading level of the EVs-ICA preparation, as determined by high-performance liquid chromatography (HPLC) analysis, was found to be between 5-8%. This study confirms the successful isolation of EVs and the successful preparation of EVs-ICA through co-incubation [123].

#### Nanocarriers

Nanocarriers are extensively employed for targeted drug delivery. A study by Ming et al. [124] showed that exosomes have impressive abilities in promoting osteoblast proliferation, particularly when transporting ICA using fetal bovine serum (FBS) as a nanocarrier. This method effectively incorporates the drug and greatly improves cell proliferation compared to using plain ICA. In another study, ICA-soymilk nanocomplexes were developed using an ultrasonic technique [125]. The results indicated a significant improvement in the solubility of ICA in water with the nanocomplexes (715–749 vs 39.03 µg/mL). Moreover, ultrahigh-performance liquid chromatography (UHPLC) results showed that the loading capacity of developed ICA-soymilk nanocomplexes ranged from 2.76-2.89%, surpassing that of ICA nanogels (2.03%) utilizing Span 80 and Tween 80 as nanocarriers [126]. Accordingly, promising candidates for enhancing the bioavailability of ICA at specific sites include nanoemulsions [127], liposomes [128], and carbon-based nanodiamonds (NDs) [129]. These various nanocarriers hold potential for improving the bioavailability of ICA at targeted locations.

#### Solid lipid nanoparticles

In recent decades, solid lipid NPs have garnered significant attention as drug delivery systems due to their effective transport of hydrophobic bioactive compounds [130]. The particles have the ability to effectively decrease the movement of bioactive substances in the lipid structure, thus avoiding merging. Consequently, improved stability is attained and the expansion of the drug into the emulsifier is minimized, ensuring a steady and uninterrupted release of the drug [131]. Liu et al. [132] conducted a study to develop lyophilized ICA stealth solid lipid nanoparticles with the aim of improving the bioavailability of ICA. The study compared them with an ICA control solution and found that the solid lipid nanoparticles greatly extended the half-life of ICA in the bloodstream and increased its relative bioavailability. Moreover, there was a significant enhancement in drug uptake observed in the kidney.

#### Absorption enhancement

Absorption enhancers are frequently used to boost the water solubility of drugs that have low solubility. This enhanced ability to dissolve bioactive compounds helps to increase their absorption in the intestine and ultimately improves their bioavailability [133]. Hydrolysis enables flavonoids to exist in both glycoside and aglycone forms. In a study by Liu et al. [134], it was found that the addition of snailase, which is comprised of a variety of enzymes such as cellulase and pectinase, can enhance the absorption of ICA. In vivo studies conducted on rats with osteoporosis demonstrated a significant reduction in the hydrolysis rates of ICA and its derivatives, resulting in decreased efficacy of these bioactive compounds [135]. To address this issue, one approach involved the packaging of ICA and snailase into enteric-coated capsules, with subsequent investigation of their pharmacokinetics in rats. The results showed that there was a significant improvement in the bioavailability of ICA, specifically in rats with osteoporosis, resulting in a 50% increase. However, they reported that no notable enhancement was observed in normal rats [134].

#### Bioactive scaffolds

Bioactive scaffolds, which serve as 3D structures for BTE, have gained significant attention in recent years [136]. These scaffolds incorporate bioactive compounds and provide essential mechanical strength for repairing bone defects [137]. Both synthetic and natural materials have been utilized for the fabrication of these bioactive scaffolds [138].

In order to promote bone tissue repair, various studies have explored the use of organic-inorganic composite scaffolds for the adsorption of ICA. A sustained release of ICA was achieved by Xie et al. [139] through the development of ICA-loaded hydroxyapatite/alginate porous composite scaffolds. In another study, Lai et al. [140] utilized 3D printing technique to fabricate a scaffold made of PLGA/β-TCP/ICA for the purpose of promoting bone formation. Reiter et al. [141] employed gelatin-coated, 3D sponge-like scaffolds for the loading of ICA. A different method that shows great regenerative potential, involves using NPs made of mesoporous bioactive glass. These particles have a high surface area and a large volume of pores. They can be customized in terms of their size and shape and are effective at carrying biomolecules for gradual release [142, 143].

In a previous study, researchers used SF as a material for scaffolding, mesoporous bioactive glass NPs as carriers for sustained release, and loaded ICA to enhance the bone growth [144]. Experimental findings demonstrated that the developed scaffolds effectively loaded and released ICA over an extended period. Furthermore, various biomaterials have been investigated to enhance the loading efficiency and bioavailability of ICA, including β-TCP [145], PLGA [146], calcium phosphate cement [147], 3D porous polydopamine/sulfonated PEEK [148], TiO_2_ nanotube/PLGA [149], titanium [150, 151], PCL/gelatin fibers [152], SF/PLCL nanofibrous scaffold [153], PLLA/PDA/chitosan [154], biphasic calcium phosphate (BCP) [155], polyvinyl alcohol (PVA)/β-TCP [156], hydroxyapatite/chitosan [157], hydroxyapatite/alginate porous composite [139], nanohydroxyapatite/polylactic acid (nHAP/PLA) [158], and PLGA/β-TCP [140].

## Biological effects of encapsulating icariin in nanoplatforms

Previous studies proved that encapsulating ICA within nanoplatforms offers significant potential for enhancing osteogenesis and bone regeneration [139, 159]. This section explores key biological impacts associated with utilizing ICA-loaded nanoplatforms, emphasizing their relevance in BTE applications.

### Osteogenic differentiation and cellular viability

**Fig. 9:**
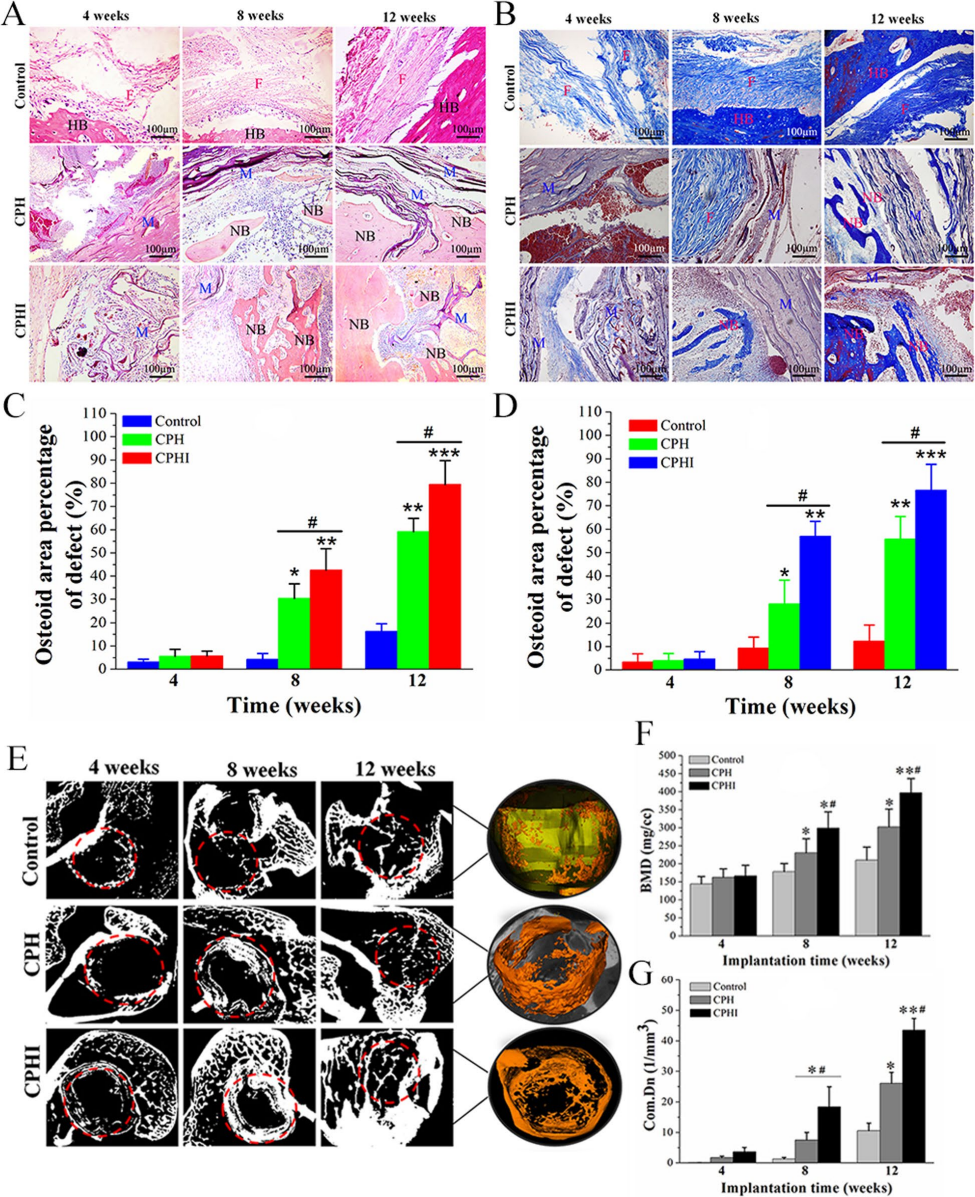
Histological staining evaluation of the newly formed bone at 4-12 weeks after implantation of CPH and CPHI scaffolds in rabbit bone defects: **A** utilizes hematoxylin and eosin (H&E) staining to showcase the new bone formation in both CPH and CPHI scaffolds; **B** on the other hand, employs Masson's trichrome staining to demonstrate the distribution of the matrix. The quantitative data from Panels **A** and **B** are displayed in **C** and **D**, respectively. These data represent the mean relative values obtained from three independent experiments (mean ± SD). Significance levels are indicated as *p < 0.05, **p < 0.01, and ***p < 0.001. The asterisk symbol (*) denotes significance compared to the defect control group, while the hashtag symbol (#) denotes significance compared to the CPH group. Moving on to **E-G**, micro-CT analysis is utilized to assess the new bone: **E** shows the micro-CT scan images indicating the level of regenerated bone tissue after 4-12 weeks, **F** represents the bone mineral density (BMD) of the regenerated bone tissue, while **G** illustrates the tissue connective density (Conn.Dn) of the regenerated bone tissue. Similar to **C** and **D**, the data in **F** and **G** are presented as mean relative values obtained from three independent experiments (mean ± SD). The significance levels are indicated as *p < 0.05, **p < 0.01, with the asterisk symbol (*) denoting significance compared to the defect control group, and the hashtag symbol (#) denoting significance compared to 8 weeks. The red dotted circles in the images represent the defect areas. Reprinted with permission from [160]

Icariin, known for its osteogenic properties, facilitates the transformation of MSCs into osteoblasts and enhances mineralization [48]. Zhao et al. [160] developed core-shell scaffolds encapsulating ICA to address tibia bone defects in rabbit models. Notably, the scaffolds demonstrated robust bone regeneration (Fig. 9), highlighting the potential of ICA-loaded core-shell composite scaffolds in BTE. They used collagen/PCL/HAP (CPH) and collagen/PCL/HAP/ICA (CPHI) scaffolds in their study. Additionally, Negrescu et al. [161] investigated the enhancement of osseointegration through modifying titanium surfaces with ICA-loaded titanium dioxide nanotubes. In vivo studies demonstrated increased bone tissue formation around the ICA-coated surfaces, indicating improved osseointegration. This approach holds promise for promoting bone tissue regeneration and reducing inflammatory responses.

### Controlled and sustained drug release

Nanoplatforms enable controlled and sustained release of ICA, optimizing its bioactivity over time [162]. Shen et al. [163] developed ICA-loaded SF/mesoporous bioactive glass NPs scaffolds for bone defects, significantly enhancing the proliferation and differentiation of BMSCs. They reported that this controlled-release scaffold system demonstrates potential for orthopedic applications, highlighting the synergy between scaffold materials and ICA-loaded NPs. Studies showed that orthopedic applications demand a vast interventions and tissue engineering products could be quiet efficient in this approach [164].

### Modulation of inflammatory responses

**Fig. 10.**
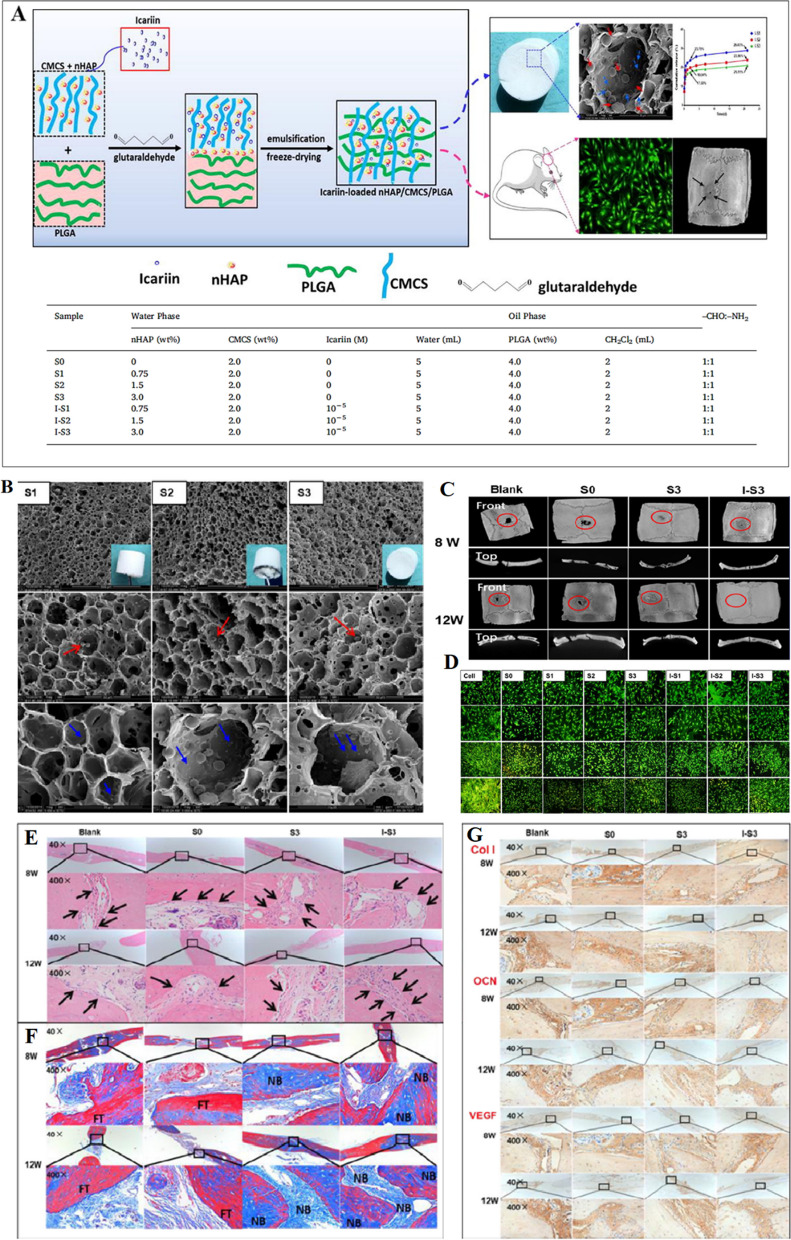
**A** Schematic illustrating the process for biomimetic fabrication of icariin-loaded nano hydroxyapatite reinforced bioactive porous scaffolds for bone regeneration. The accompanying table provides a detailed delineation of the component values within each respective sample; **B** SEM images at magnifications of 500×, 2000×, and 5000×; **C** μ-CT images of the front and top scanning of SD rats calvaria bone defects; **D** Giemsa and AO/EB staining of osteoblasts cultured with leaching liquors of S0, S1, S2, S3, and I-S1, I-S2, I-S3 scaffolds. AO/EB staining at 100× magnification for 1, 3, 5, and 7 days using an AO/EB staining kit. Osteoblasts cultured with DMEM/F-12 media with 10% FBS and 1% P/S served as the control group; **E** HE staining; **F** Masson's trichrome staining; **G** immunohistological staining of specific bone marker (Col, OCN, VEGF) analysis of SD rats calvaria bone defects at magnifications of 40× and 400× after 8 W and 12 W implantation of three different scaffolds: S0, S3, I-S3 scaffolds, and non-treated (control). The arrowheads indicate the boundary between nascent bone and host bone, and the FT, NB respectively indicate the fibrous tissue and nascent bone. All images are reprinted with permission from [166]

Icariin's anti-inflammatory effects, when encapsulated in nanoplatforms, can mitigate inflammation at the site of interest [165]. Hu et al. [166] developed biomimetic scaffolds loaded with ICA and nHAP, exhibiting significant potential for bone repair and regeneration (Fig. 10). These scaffolds effectively attenuated inflammatory reactions in vivo, underscoring their therapeutic value in BTE [166].

### Enhancement of cellular adhesion and migration

Icariin-loaded nanoplatforms support cellular attachment and migration, crucial for tissue regeneration [160, 161]. NFs, mimicking the natural ECM, offer a 3D environment that promotes cell adhesion and migration [31]. Shen et al. [163] demonstrated improved BMSC proliferation on ICA-loaded SF/mesoporous bioactive glass NPs scaffolds, while Hu et al. [166] showcased enhanced osteoblast activity on ICA-loaded nHAP/CMCS/PLGA scaffolds (Fig. 10E-G). These findings underscore the potential of ICA-loaded scaffolds in promoting cell adhesion and bone repair.

### Synergistic interplay with signaling pathways

The incorporation of ICA within nanoplatforms potentiates its inherent effects and creates a synergistic interplay with cellular signaling pathways [47, 167]. The development of a multifunctional scaffold is vital in addressing the difficult regeneration of osteoporotic bone defects, which includes problems with bone regeneration and excessive bone resorption. To address this demand, Song et al. [159] introduced an ICA-loaded strontium/BCP scaffold stimulating both bone formation and resorption through intricate signaling pathways. These multifunctional scaffolds offer promising clinical treatment strategies for repairing osteoporotic defects.

Encapsulating ICA in nanoplatforms augments its multifaceted effects on bone regeneration. From directing cellular differentiation to controlling inflammation and optimizing drug release, these platforms provide a powerful toolkit for fostering optimal osteogenesis. The next section will explore the physical effects of encapsulating ICA in nanoplatforms, shedding light on the significance of parameters such as nanofiber and nanoparticle diameter in shaping the therapeutic outcomes.

## Physical effects of encapsulating icariin in nanoplatforms

The encapsulation of bioactive compounds, such as ICA, within nanoplatforms, particularly NFs and NPs, revolutionizes strategies for promoting osteogenesis and bone regeneration. This section delves into the significant physical effects facilitated by encapsulating ICA within these nanoplatforms.

### Nanofiber diameter and morphology

Nanofibers offer precise control over diameter, influencing cellular responses and tissue integration [35, 168]. Tuning nanofiber diameter mimics the natural ECM, enhancing cellular adhesion, proliferation, and differentiation [169, 170]. Control over ICA release from NFs, modulated by diameter, ensures sustained and localized therapeutic effects, optimizing bone regeneration [163, 171]. Studies showed that various fiber diameters have been achieved through encapsulating the ICA in NFs. However, encapsulating ICA resulted in a small increase in the NF diameter.

### Nanoparticle size and surface characteristics

Nanoparticles exhibit size-dependent properties crucial for cellular uptake and interactions [35, 172]. Smaller NPs enhance ICA bioavailability within target cells, while surface characteristics influence cellular interactions, potentially amplifying therapeutic effects [173–175]. Studies showed that the size and surface characteristics of NPs changed with encapsulating ICA, while its increase or decrease was dependent on the material composition and manufacturing technology.

### Synergistic effects of nanofibers and nanoparticles

The combined use of NFs and NPs as dual carriers maximizes therapeutic impact [176, 177]. Integrating controlled release and precise targeting, this synergy optimizes osteogenic responses, fostering accelerated bone regeneration [178, 179]. Ultimately, encapsulating ICA within NFs and NPs represents a transformative approach in BTE. The meticulous control over nanofiber diameter and nanoparticle characteristics offers novel avenues for tailored therapeutic interventions. The synergy between these nanoplatforms sets the stage for optimized osteogenesis, shaping the future of regenerative medicine.

## Applications in bone tissue engineering

The maturation of osteoblasts and the formation of bones can potentially be improved by ICA, showcasing its potential in treating osteoporosis [180]. It boosts ALP function and raises the expression of receptor activator of nuclear factor κ-B ligand, resulting in enhanced bone formation. Moreover, it impacts the BMP-2/Smad4 pathway, increasing the expression of BMP-2 and Smad4, which helps with the creation of calcified nodules in bone [181].

Several studies have demonstrated the osteogenic effects of ICA, which can be attributed to its hormone-like function. It has been found to induce the expression of BMP-2 and BMP-4 mRNA in osteoblasts and significantly upregulated Osx at low doses [182]. Additionally, ICA promotes bone formation by stimulating the expression of pre-osteoblastic genes like Osx, RUNX2, and collagen type I [183].

**Fig. 11.**
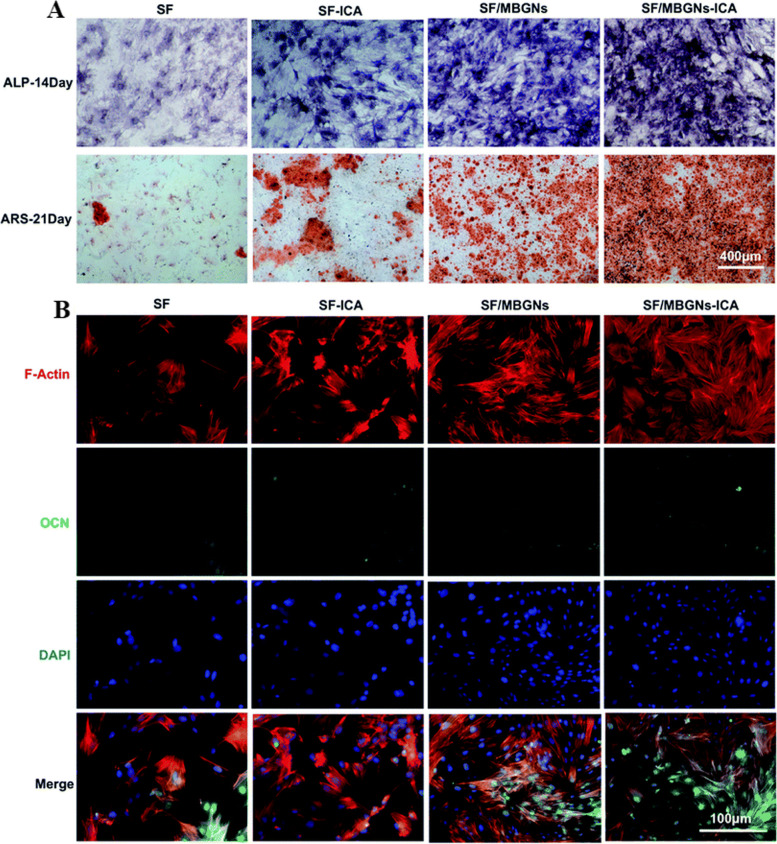
**A** Osteogenesis of BMSCs assessed by ALP staining (7 d) and Alizarin Red staining (21 d); **B** Immunofluorescence assays for OCN expression (14 d) with DAPI-stained nucleus (blue) and OCN staining (green). Reprinted with permission from [163]

Shen et al. [163] utilized SF as a scaffold and mesoporous bioactive glass NPs as a carrier for ICA. Cell viability assays revealed that ICA had minimal effects on cell viability and did not induce cytotoxicity. Furthermore, the developed scaffolds with and without ICA loading showed significantly higher ALP staining and ALP expression compared to other groups (Fig. 11A). After 21 days of cell induction, the ICA-loaded SF/mesoporous bioactive glass NPs group exhibited more calcium nodules, as evidenced by Alizarin Red staining (Fig. 11A).

The quantified results confirmed that the slow release of ICA from NPs and the presence of mesoporous bioactive glass NPs synergistically promoted osteogenic differentiation. In contrast, pure SF scaffolds had no significant effect on osteogenic differentiation. Immunofluorescence staining for OCN at 21 days of induction culture showed similar results to the calcium nodule staining. The expression levels of OCN were significantly higher in the mesoporous bioactive glass NP group and ICA-loaded SF/mesoporous bioactive glass NP group compared to the other groups (Fig. 11B).

Chen et al. [78], explored the osteogenic potential of ICA for bone health. Their study demonstrated that ICA enhanced BMP-2-induced osteoblastic differentiation of C2C12 cells, indicating its positive impact on osteogenesis. They elucidated that ICA's effect involved activating the cAMP/PKA/CREB signaling axis, with potential implications for enhancing the therapeutic efficacy of BMP-2 and offering a cost-effective alternative for promoting osteogenesis.

**Fig. 12:**
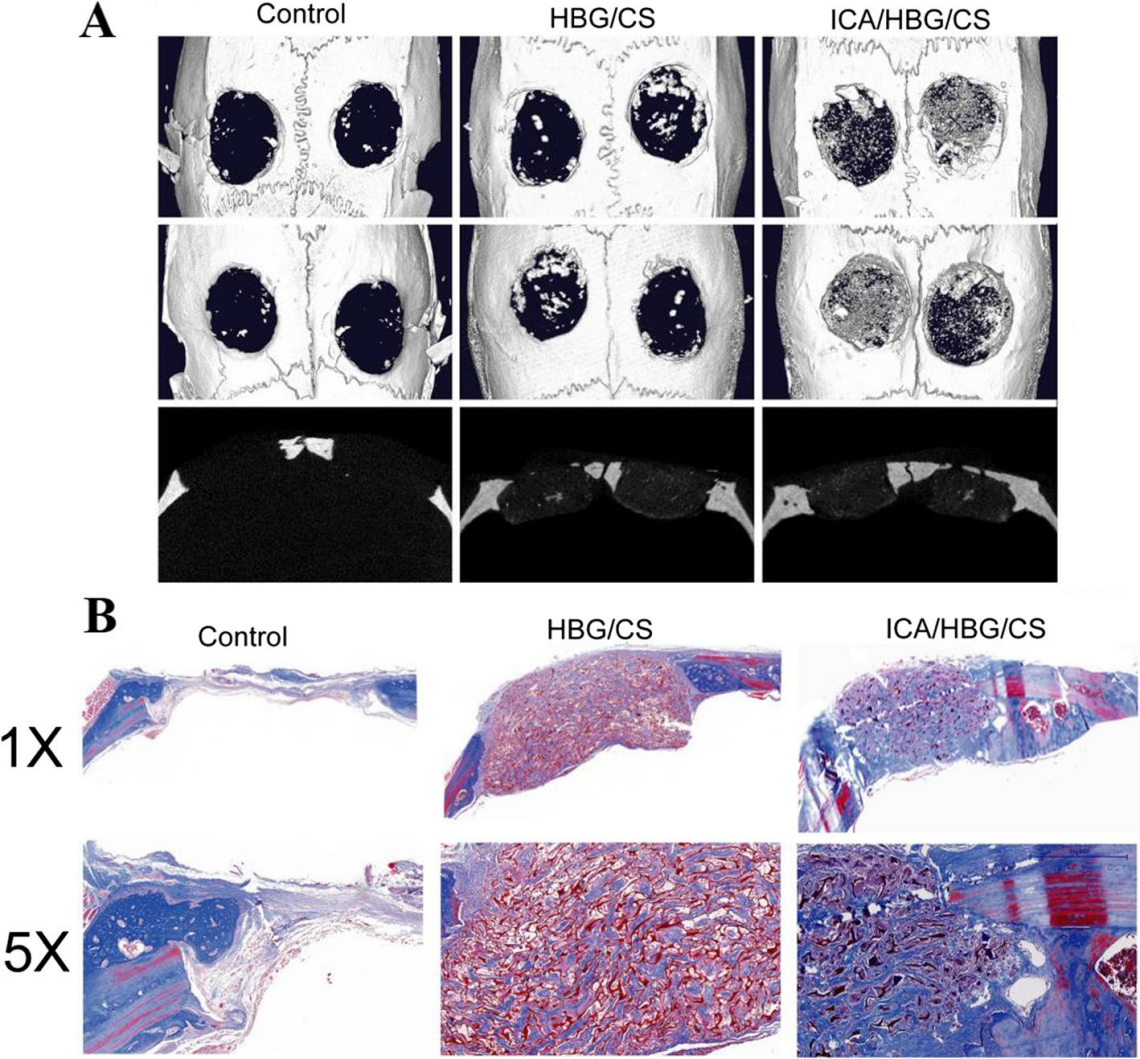
**A** Micro-CT images depicting the response of critical-sized rat calvaria defects to a blank control, HBG/CS, and ICA/HBG/CS scaffolds over a 12-week period; **B** Histomorphological evaluation of the three groups using Masson's trichrome staining to assess the formation of newly generated bone (blue) and collagen components (red). Reprinted with permission from [184]

In a study, therapeutic scaffolds loaded with ICA within hollow bioglass/chitosan (ICA/HBG/CS) were developed for the treatment of critical-sized bone defects [184]. The expression of osteogenic-related genes and proteins was significantly increased by the released ICA drugs from the scaffolds. Furthermore, the 3D reconstructed micro-CT images of critical-sized rat calvaria defects showed limited new bone tissue formation in the blank control group after 12 weeks. However, both ICA/HBG/CS and HBG/CS scaffolds accelerated the formation of new bone tissues, with the former showing a greater amount of newly formed bone. This was supported by Masson's trichrome staining, which demonstrated a higher presence of new bone in the ICA/HBG/CS group compared to the HBG/CS group, indicating that the released ICA drugs promoted mineralization of osteogenic tissue (Fig. 12).

In another study, Choi et al. [185] aimed to enhance the osteogenic capacity of NDs by developing ICA-functionalized nanodiamonds (ICA-NDs). The ICA-NDs were tiny spherical particles and achieved a continuous release of ICA for a period of over 4 weeks. In experiments conducted on MC3T3-E1 cells, it was noticed that cells treated with ICA (50 μg)-NDs displayed a notable increase in markers associated with bone formation, such as ALP activity, calcium content, and gene expression levels of osteogenic-related markers (ALP, RUNX2, COL1A1, and OPN), in comparison with cells treated with ICA (10 μg)-NDs or unaltered NDs. These findings indicated the potential of ICA-NDs to enhance osteogenic capacity, making them promising candidates for applications in BTE.

Also, encapsulating ICA within NFs holds significant promise for promoting osteogenesis and bone regeneration. The unique morphology and diameter of NFs, which can be precisely controlled, play a crucial role in influencing cellular responses and tissue integration. By replicating the structure of the natural ECM, NFs promote improved cellular adhesion, proliferation, and differentiation. The controlled release of ICA from these NFs can provide sustained and localized therapeutic effects, optimizing the promotion of osteogenesis and bone regeneration.

Several studies have demonstrated the potential of ICA-loaded NFs in BTE. For instance, Hu et al. [166] developed innovative biomimetic scaffolds for BTE by incorporating ICA and nHAP using hybrid techniques. These scaffolds mimicked the natural bone structure, leading to improved mechanical properties and accelerated tissue repair. In vivo experiments exhibited strong biocompatibility and enhanced osteoblast activity, confirmed by histological analysis. This research underscores the potential of ICA-loaded nHAP/PLGA scaffolds as a promising avenue for promoting cell adhesion and bone repair, while also mitigating inflammatory reactions.

**Fig. 13:**
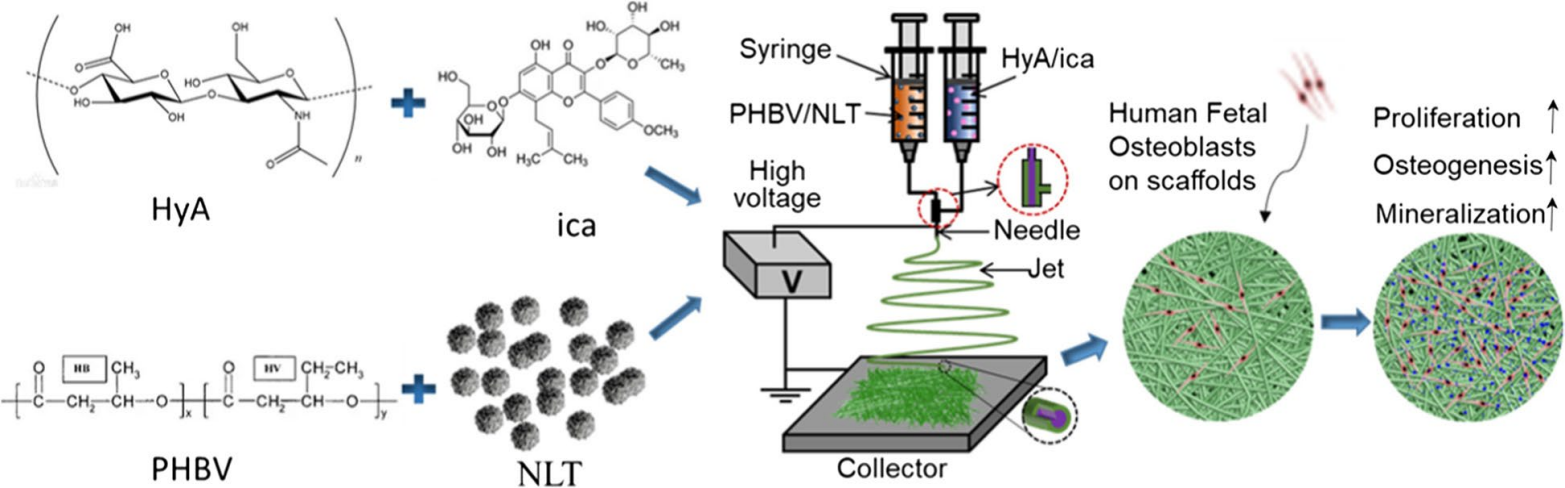
The fabrication process and application of PHBV/NLT-HyA/ICA coaxial nanofiber scaffold in BTE. Reprinted with permission from [186]

Zhang et al. [186] introduced a novel approach for BTE by developing an electrospun nanofiber scaffold. The scaffold consisted of a core-shell structure, with the core composed of HA loaded with ICA, and the sheath comprised of poly-3-hydroxybutyrate-co-3-hydroxyvalerate (PHBV) loaded with perovskite (Na_2_La_2_Ti_3_O_10_, NLT) NPs (Fig. 13). They reported that the developed ICA-loaded PHBV/NLT-HA nanofiber scaffolds demonstrated remarkable characteristics, including a stable network structure, excellent surface hydrophilicity, and superior mechanical properties [186]. Significantly, the viability, proliferation, and maturation of human fetal osteoblasts were improved by the controlled release of ICA from the coaxial NFs. Additionally, the combination of NLT loading and ICA release resulted in enhanced ALP activity and calcium content, demonstrating the great potential of this innovative scaffold for BTE. The study also suggested the need for further research using animal experiments [186].

**Fig. 14.**
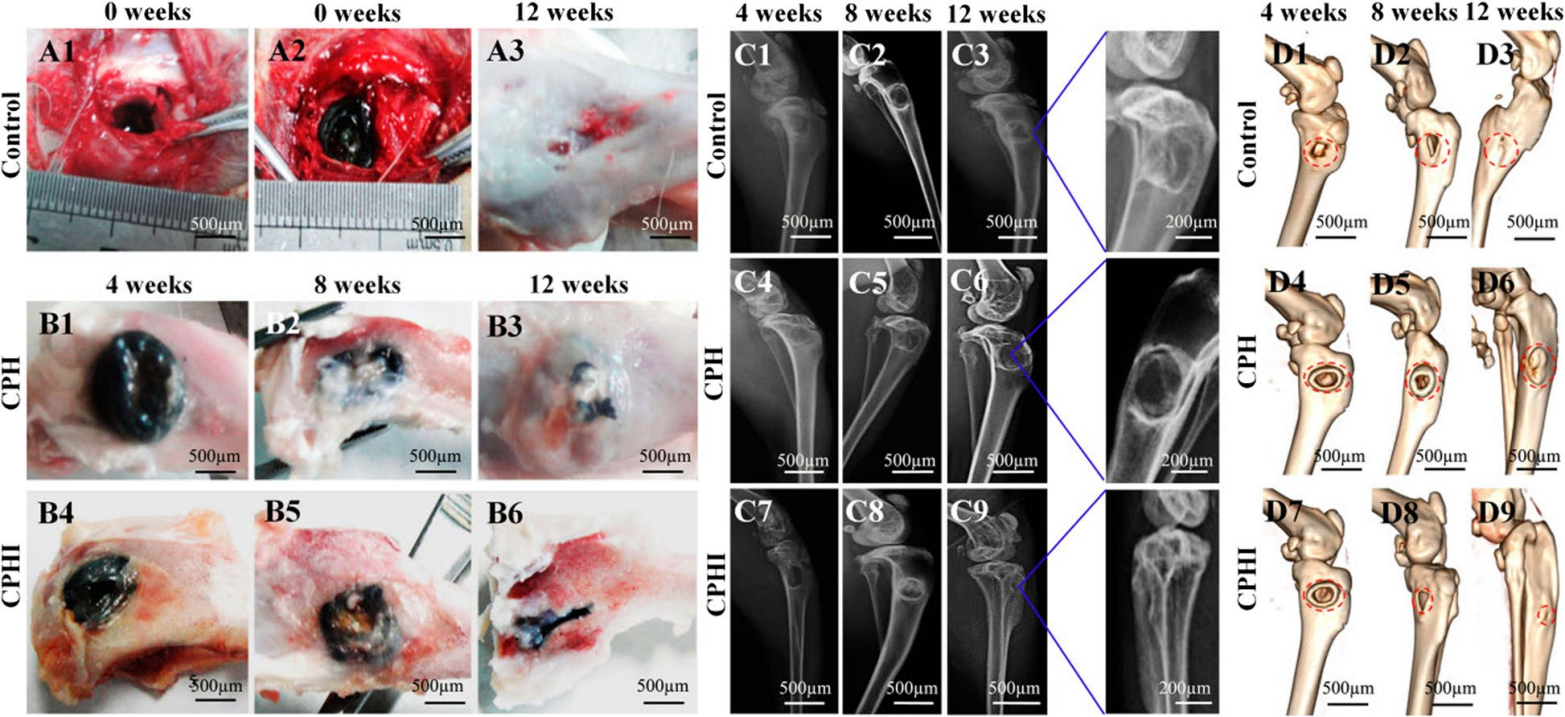
**A** Photographs illustrating the surgical implantation procedure of CPH and CPHI scaffolds in rabbit bone defects (A1 and A2). Additionally, (A3) shows the defect control group covered by connective tissue after 12 weeks. **B** The progression of new bone formation in the CPH and CPHI groups over 4, 8, and 12 weeks (B1-B6). **C** Present representative X-ray images evaluating the level of regenerated bone tissue after 4-12 weeks (C1-C9). **D** reconstruction images revealed the distinct reparative effects of the CPH and CPHI scaffolds after 4-12 weeks (D1-D9). Red arrows indicate bone density, red dotted circles highlight defect areas, and red arrows denote areas of dense bone regeneration. Reprinted with permission from [160]

Similarly, Zhao et al. [160] introduced a novel method for BTE using core-shell scaffolds that can carry drugs. They produced these scaffolds by combining electrospinning and freeze-drying techniques, incorporating chitosan microspheres loaded with ICA into collagen core scaffolds. As a result, composite scaffolds consisting of collagen, PCL, and HAP shell materials were formed. The cross-linked developed scaffolds possessed exceptional physical, chemical, and mechanical properties, which facilitated the attachment and growth of rat bone marrow MSCs. In vivo evaluations using rabbit models with tibial plateau defects demonstrated significant new bone formation on the developed scaffolds, highlighting their potential for effective bone regeneration and repair [160]. The tibial plateau bone defects in rabbits treated with various scaffold materials are shown in Fig. 14A and B before and after 4-12 weeks of implantation. The images depict the defect control group (A1-3) and the PCL/collagen/HAP and ICA-loaded PCL/collagen/HAP scaffolds (B1-6).

Comparatively, the ICA-loaded PCL/collagen/HAP scaffolds demonstrated a significant reduction in the size of bone defects compared to the scaffolds without ICA and the defect control group. Instead of new bone tissue, connective tissue covered the defect area in A3. According to the X-ray examination results in Fig. 14C, there was no evidence of low-density bone regeneration in the bone defect areas four weeks after surgery. 3D CT reconstruction using Fig. 14D was performed to visualize the bone defects in the tibial plateau after 4-12 weeks of implantation. The reconstructed 3D CT images directly showed the presence of newly formed bone in the defect areas. After 12 weeks of implantation, a larger amount of newly formed bone was observed in the area where ICA-loaded PCL/collagen/HAP scaffolds were implanted compared to scaffolds without ICA (D9). The findings suggest the enhanced efficacy of ICA-loaded scaffolds in promoting bone regeneration.

Liu et al. [89] implemented a novel method to create a 3D scaffold with ICA for bone regeneration. This approach involved the use of coaxial electrospinning and electrospraying techniques. The scaffolds loaded with the drug displayed strong mechanical properties and stability. The controlled release of ICA within the scaffold facilitated increased ALP activity and deposition of ECM minerals in the MSCs. Consequently, this created a beneficial environment for osteogenic applications in BTE.

Zhao et al. [94] explored a novel electrospinning method to create a composite scaffold of PLGA loaded with ICA. Demonstrating the slow and gradual release of ICA from the scaffold suggests its potential for inhibiting osteoarthritis progression. The scaffold displayed remarkable biocompatibility and encouraged the production of the ECM, preserving the functional structure of articular cartilage and preventing the resorption of subchondral bone trabeculae. These findings indicate the therapeutic potential of the scaffold in treating osteoarthritis. In summary, ICA-loaded NFs have shown significant potential in promoting osteogenesis and bone regeneration. They offer a promising avenue for innovative therapeutic strategies, capitalizing on the distinct attributes of NFs to augment the delivery and accessibility of bioactive agents.

To advance BTE, researchers have developed biomimetic scaffolds using a combination of water-soluble carboxymethyl chitosan (CMCS) and oil-soluble PLGA through an emulsion template method. This innovative approach involved the addition of nHAP and ICA to the hybrid scaffolds, significantly enhancing their mechanical properties and bioactivity in vitro. Notably, in vivo osteogenesis studies conducted on the ICA-loaded nHAP/CMCS/PLGA scaffolds, particularly the 3.0 wt.% nHAP/CMCS/PLGA scaffold and the ICA-loaded 3.0 wt.% nHAP/CMCS/PLGA scaffold, demonstrated an effective enhancement in osteoblast adhesion, proliferation, and differentiation [166]. These findings underscore the considerable potential of ICA-containing scaffolds for bone repair and regeneration applications.

In a previous study, a 3D printing technique was used to fabricate alginate dialdehyde-gelatin hydrogel reinforced with mesoporous silica-calcium NPs and loaded with ICA [187]. Biocompatibility tests demonstrated enhanced osteoblast proliferation, adhesion, and differentiation, attributed to the favorable properties of developed hydrogel, the bioactivity of the NPs, and the supportive effect of ICA on cell proliferation and differentiation. However, it should be noted that the concentration of ICA plays a crucial role in its effects, with low concentrations stimulating BMSCs proliferation and higher concentrations exhibiting cytotoxicity [188]. Therefore, it is important to modify the release profile and kinetics of ICA. This can be done by loading it into an appropriate carrier in a customized manner.

Additionally, ICA has demonstrated potential in the post-infection treatment of bone infection. Osteogenic cells and bone mass can be adversely affected by high concentrations of vancomycin, a commonly used antibiotic for controlling bone infections [189]. However, the negative effects of vancomycin were counteracted when combined with ICA treatment. ICA exhibited regulatory effects on the cell cycle, stimulated ALP activity, and upregulated the BMP-2/ RUNX2 and OPG/RANKL pathways [190]. Zhang et al. [191] conducted a study with the aim of evaluating the efficacy of ICA in a rabbit model of bone infection treated with vancomycin, as well as investigating its effect on the proliferation of osteoblasts induced by vancomycin.

**Fig. 15.**
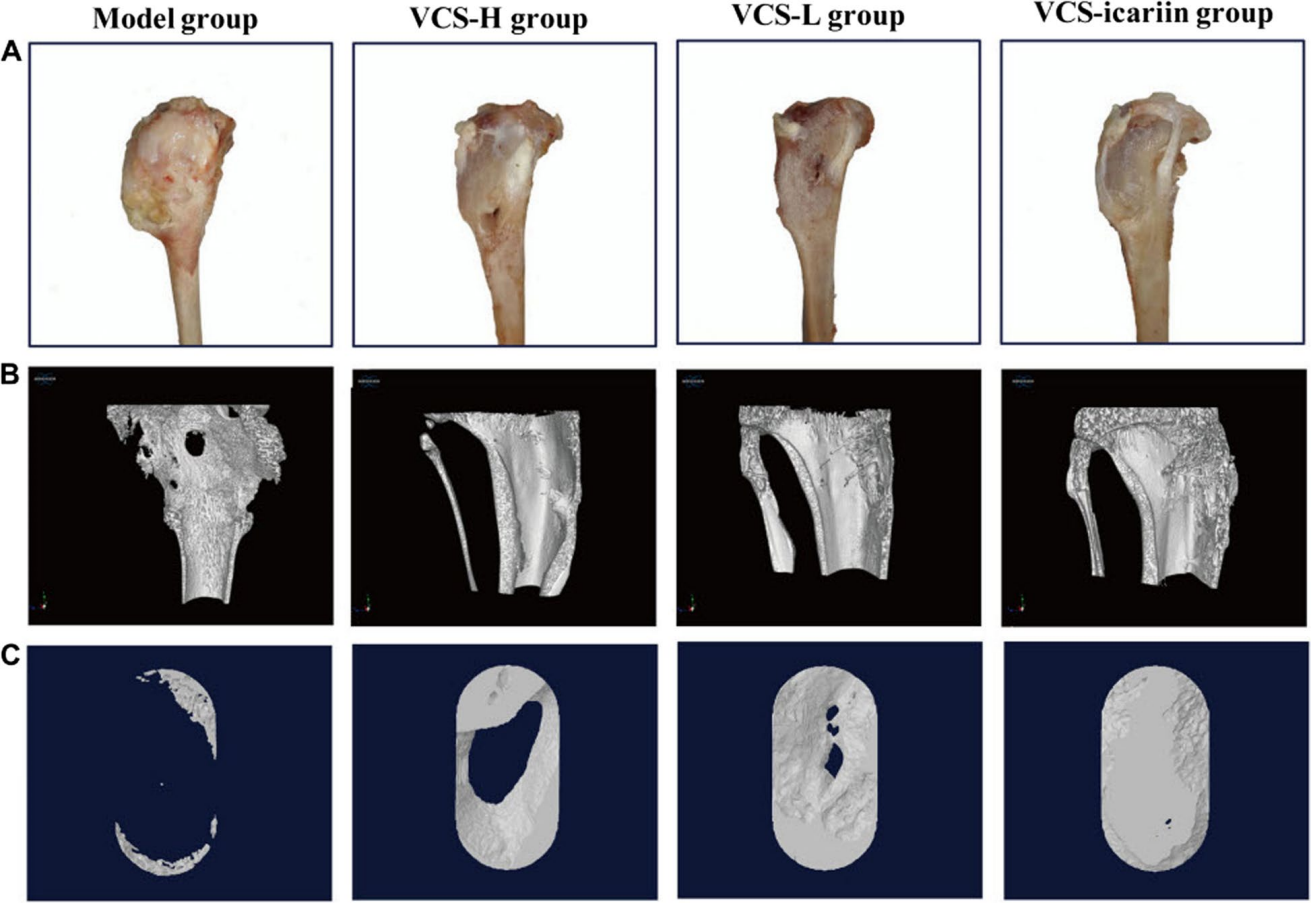
**A** The visual representation of rabbit tibias after 8 weeks of treatment with VCS-L, VCS-H, and VCS-icariin; **B** 3D reconstruction illustrating the bone defects in the tibia of rabbits in the bone infection model group following 8 weeks of treatment with VCS-L, VCS-H, and VCS-icariin; **C** 3D reconstruction displaying the bone defect in the region of interest in the model, VCS-L, VCS-H, and VCS-icariin groups (**C**). Reprinted with permission from [191]

According to Fig. 15A, after 8 weeks of treatment, rabbits in the model group displayed a large number of infected sinuses with the presence of white and yellow pus overflowing from the wounds. In comparison, the vancomycin/ICA group had the smallest bone defects among the three treatment groups, while the high-dose vancomycin group had the largest defects. Micro-CT results, presented in Fig. 15B and C, highlighted evident bone defects in the model group through 3D reconstruction figures of the tibia and the region of interest.

However, the low-dose vancomycin (VCS-L), high-dose vancomycin (VCS-H), and vancomycin/ICA (VCS-icariin) groups exhibited significantly reduced defect areas, with the vancomycin/ICA group showed the most improved bone mass compared to the other groups. Moreover, in the model group, the ratio of bone volume/tissue volume (BV/TV) and bone morphogenetic protein (BMD) were measured at 10.34 ± 1.62% and 0.32 ± 0.03 g/cm^2^, respectively. However, the vancomycin/ICA treatment markedly increased BV/TV to 75.33 ± 4.25% and BMD to 1.26 ± 0.04 g/cm^2^, which were significantly higher than the indicators in other groups. These results suggested that high local concentrations of vancomycin influenced bone healing after anti-infection treatment, but ICA effectively promoted bone repair [191].

Autophagy is a crucial cellular process involved in the elimination of cellular waste and damaged components, contributing to various physiological and pathological processes [192]. However, aging is associated with a decline in autophagic degradation, leading to the accumulation of damaged cellular components and contributing to age-related cellular dysfunction [193]. The activation of autophagy, particularly through mTORC1 inhibition, can enhance the degradation of aged cellular components, thereby promoting cellular function during aging [194]. Consequently, there is a growing interest in using exogenous drugs to activate autophagy and rejuvenate senescent BMSCs and alleviate inflammation in senescent macrophages as an innovative strategy for treating osteoporosis [195]. ICA, at an appropriate concentration, has been found to promote bone formation, inhibit bone resorption, and activate the BMP and Wnt/β-catenin signaling pathways without any adverse effects [196].

**Fig. 16:**
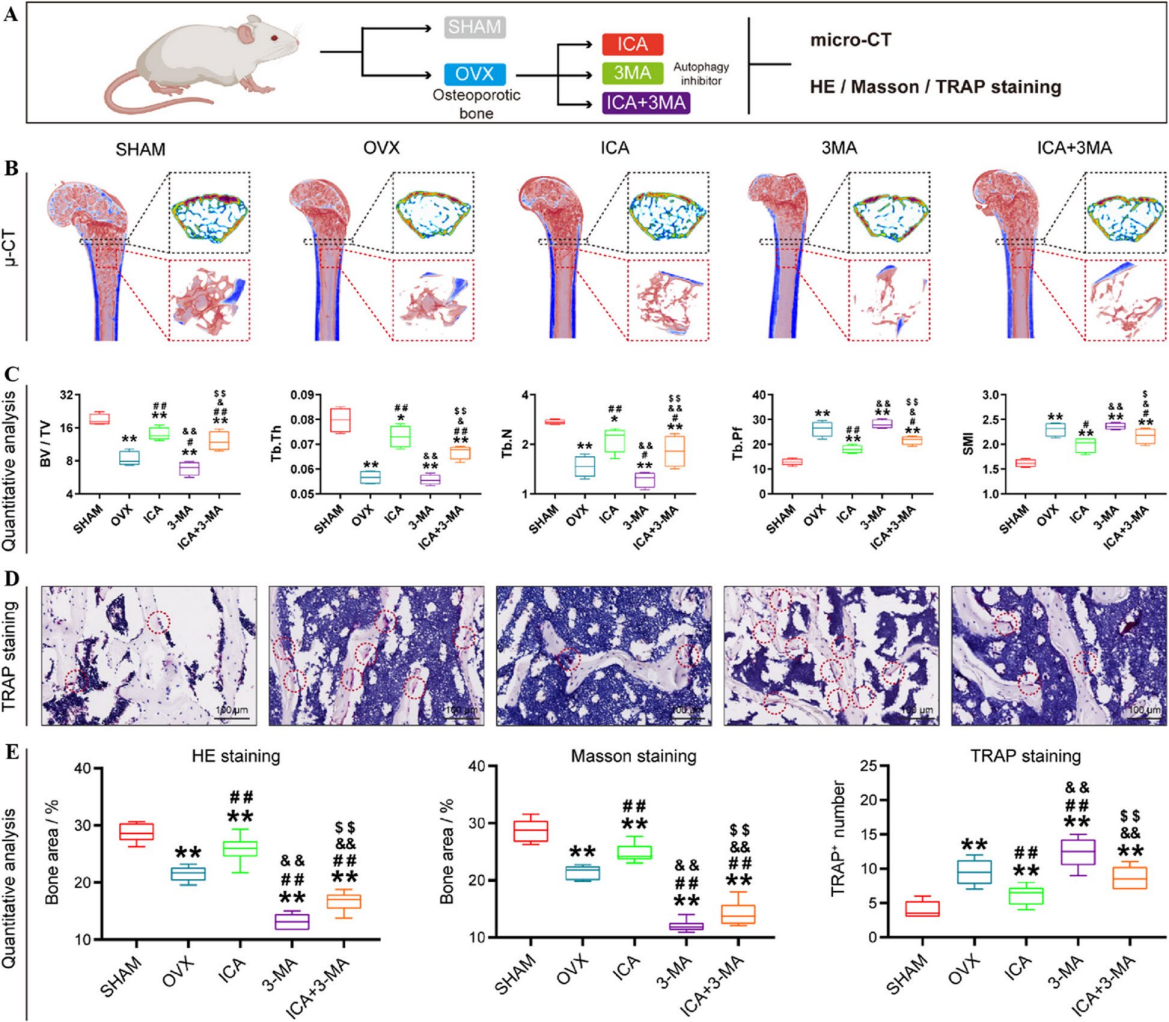
In vivo attenuation of osteoporosis following ICA stimulation. **A** Schematic representation of animal tests; **B** Representative 3D micro-CT images illustrating bone tissue, with magnified areas showing cross-sections in 2D and partial bone volume in 3D; **C** Quantitative assessment of bone tissue parameters (BV/TV, Tb⋅Th, Tb. N, Tb. Pf, and SMI) (*n* = 6); **D** TRAP staining of bone tissue across the groups; **E** Quantitative analysis of pathological slices (*n* = 6). *p < 0.05 vs. Sham, #*p* < 0.05 vs. OVX, &p < 0.05 vs. ICA, $p < 0.05 vs. 3-MA, **p < 0.01 vs. Sham, ##*p* < 0.01 vs. OVX, &&*p* < 0.01 vs. ICA, $$*p* < 0.01 vs. 3-MA. Reprinted with permission from [197]

In a study by Bai et al. [197], the effects of ICA on senescent BMSCs and senescent macrophages under conditions of osteoporosis and inflammation were investigated (Fig. 16A and B). The study demonstrated that ICA effectively alleviated bone loss in osteoporosis by activating autophagy. Micro-CT imaging revealed a significant reduction in bone mass in the ovariectomy group, indicating the successful establishment of an osteoporosis model.

TRAP staining indicated increased osteoclast activity in the ovariectomy group, which was reduced by ICA treatment. Furthermore, inhibition of autophagy by 3-methyladenine (3MA, an autophagy inhibitor) led to a decrease in bone volume, emphasizing the crucial role of autophagy in bone formation. Although bone mass increased with the combination of ICA and 3-methyladenine, it was significantly lower than with the ICA treatment alone, highlighting the regulatory role of autophagy. These findings provided evidence that ICA effectively alleviates bone loss in osteoporosis by activating autophagy (Fig. 16C-E) [197].

A comprehensive comparative analysis of the effects of ICA on various bone tissue properties is presented in the Table 1, highlighting its potential as a therapeutic agent for conditions related to bone health.

**Table 1:** An easy-to-understand comparison of the effects of ICA on various bone tissue characteristics, emphasizing its potential as a therapeutic agent for bone-related conditions

Compound	Fabrication method	Dosage	Types of experiment	Major findings	**Ref.**
ICA-PDA@SPEEK	Polymerization	16, 32, 64 µg ml^-1^	In vitro, in vivo	Regulating cytokine secretion by macrophages increased the proportion of M2-polarized macrophages, facilitated osteogenesis, and impeded osteoclast differentiation	[148]
SF/MBGNs-ICA	Freeze-drying	120 µg ml^-1^	In vitro	Increased the expression level of OCN, ALP, RUNX2, and OPN	[163]
ICA-Ti particles	MC3T3-E1 cells were pretreated with icariin and then incubated with Ti particles	10^-8^ M	In vitro	Increased ALP activity, accelerated matrix mineralization, and upregulated the levels of BMP-2, RUNX2, OCN, and miR-21-5p	[198]
ICA-BBL@HA	Thin-film dispersion and mechanical extrusion	-	In vitro, in vivo	Reestablish rat bone microarchitecture afflicted by osteoporosis symptoms	[128]
ICA@PCL-Gelatin	Electrospinning	0.5, 2.0, 5.0 wt.%	In vitro, in vivo	Regulation of the TGF-β and Smad pathways had an impact on adhesion formation in vivo by inhibiting fibroblast proliferation and decreasing collagen synthesis	[90]
ICA-SF/PLCL	Coaxial electrospinning	10^−5^ mol/L	In vitro, in vivo	Significantly promoted the osteogenesis of BMMSCs in vitro and repair bone defect in vivo	[99]
VCS-ICA	-	10.86 mg/kg/day	In vitro, in vivo	Promoted bone formation by up-regulating BMP-2/ RUNX2 and OPG/RANKL pathways	[191]
PLLA/CS-PDA/ICA/DFO	Electrospinning and thermally induced phase separation	0.02 mg/mL	In vitro	Enhanced cell adhesion, proliferation, osteogenic differentiation and mineralization of MC3T3-E1; significantly promoted the growth and expression of angiogenic-related factors of HUVECs	[93]
ICA-loaded hyaluronic acid/chitosan (HA/CS)	Phase-transited lysozyme (PTL) and layer-by-layer (LbL) self-assembly system	0.5×10^-3^, 1×10^-3^, 2×10^-3^ mol/L	In vitro, in vivo	Increased osteoblast ((MC3T3-E1) proliferation at low doses and aligned calcified bone-like collagen matrix	[151]
ICA-PLGA@TiO_2_	Coating	2 × 10^-3^ mol/L	In vitro, in vivo	Sustained release of icariin until two weeks; improved cell adhesion, proliferation, and differentiation of MC3T3-E1	[199]
ICA-PD@TiO_2_	Electrochemical anodization	500 µM	In vitro, in vivo	The acute inflammatory response was suppressed, resulting in a decrease in the fibrotic capsule around the implant and an increase in the thickness of newly formed bone tissue, first at 1 month and then at 3 months after implantation	[161]
ICA-TIO_2_-ASP@PLGA	Coating	1.15 mg/mL	In vitro	At the same time, the modified surface provided the ability to modulate the immune response in macrophages and promote bone formation in osteoblasts	[200]
ICA/β-CD-conjugated alginate	Chemical reaction of carboxylated CD with aminated ALG and ICA inclusion	1, 5, 10 µM	In vitro	The osteogenic ability of MC3T3-E1 cells was improved through the release of ICA from the inclusion nanocomplex, which resulted in increased levels of ALP, calcium, and the expression of OCN and OPN	[201]
(β-CD-ALG)					
ICA-MTZ@CPC	Sol-gel	2 mg	In vitro	A significant decrease in the growth activity of planktonic porphyromonas gingivalis and bacterial biofilms; promoted the expression level of RUNX2 and BSP	[147]
ICA-NDs	Sol-gel	10 and 50 µg	In vitro	The sustained release of ICA from the NDs increased the expression of early osteogenic-related genes (ALP and RUNX2) and late osteogenic-related genes (COL1A1 and OPN)	[185]
ICA-MSN@ADA-GEL	3D printing	1000 µg/mL	In vitro	Enhanced osteoblast proliferation, adhesion, and differentiation of MC3T3-E1 cells	[187]
PHBV/NLT-HyA/ICA nanofiber	Coaxial electrospinning	ICA with a mass ratio of 1:1 was dissolved into water/ethanol (1:1, v/v) to generate the concentration of 2.4% (w/v)	In vitro	The viability and growth of human fetal osteoblasts (HFOBs) were greatly enhanced, along with their development into mature cells	[186]
PCL/Fe_3_O_4_/ICA	Electrospinning and depressurization of subcritical CO_2_ fluid	0.1 w/v%	In vitro, in vivo	Greatly promoted cell viability, cell penetration, collagen deposition, and angiogenesis	[91]
ICA-CPC tablets	Freeze drying	1 mg	In vitro, in vivo	Increased osteogenic differentiation; Accelerated bone regeneration at 4 and 6 weeks after transplantation	[202]
ICA/HBG/CS	Freeze-drying	10, 25, 50 µM	In vitro, in vivo	The expression levels of osteogenic-related genes (COL1 and RUNX2) and osteogenic-related proteins (ALP and p-Smad1/5) were significantly increased; the formation of new bone tissues was significantly accelerated	[184]
ICA-mHNT@CS-GP	Sol-gel	-	In vitro	Mesenchymal stem cells experience improved cell proliferation and bone differentiation, while the initial burst release of ICA is decreased and entrapment efficiency and loading capacity are increased	[203]
ICA-loaded nHAP/CMCS/PLGA	Emulsion polymerization	10−5 M	In vitro, in vivo	Osteoblast adhesion, proliferation, and differentiation were enhanced by improving mechanical properties and in vitro bioactivity	[166]
ICA@PLGA/PCL-nHAP	Emulsion solvent evaporation and 3D printing	250 μL	In vitro, in vivo	The ICA released facilitated the differentiation of MC3T3-E1cells into bone cells and promoted the healing of calvarial bone	[204]
PLGA/TCP/ICA	3D printing	0.16% (the mass ratio of PLGA to TCP to icariin was (80:20:0.16), 0.32% (80:20:0.32), 0.64% (80:20:0.64)	In vitro, in vivo	The SAON rabbit experienced improved angiogenesis in the implanted region due to increased mechanical support, stable icariin release from the scaffold, and enhanced mechanical properties of new bone tissues	[140]
IC/Sr-BCP	Solidification and H_2_O_2_ gas foaming	1.5 μmol	In vitro, in vivo	The co-delivery system has the potential to enhance osteogenesis by increasing the levels of osteogenesis-related proteins such as alkaline phosphatase, osteocalcin, and BMP-2. Additionally, it hinders osteoclastogenesis	[159]
FBS EXO-ICA	Ultracentrifugation	1 mg.ml^-1^	In vitro	Promoted the proliferation of osteoblasts and bone regeneration	[124]
BG/Sr/ICA	Sol-gel	1 mg.ml^−1^	In vitro, in vivo	Improved the osteogenic potential presented by BMMSCs from rats with osteoporosis	[205]
PCL/β-TCP/ICA	Extrusion-based 3D printing	0.16, 0.32, and 0.64% of the total stent mass	In vitro	The expression of genes specific to osteoblasts was greatly increased	[206]
PCL/Gelatin/ICA	Electrospinning		In vitro	Increased OCN and type collagen I (COL I) expression in MC3T3-E1cells	[88]
PGCL/HA/dECM/ICA	Emulsion-solidification	mass ratio (90:10:0.32)	In vitro, in vivo	Synergistically enhanced the migration and osteogenic differentiation of BMSCs	[145]
PLA/nHAC/ICA	Dyeing	20–30 µg/ml	In vitro, in vivo	By increasing the levels of BMP-2 and OPG proteins, it enhanced the growth of osteoblasts and promoted their proliferation. Additionally, it also stimulated the expression of BMP-2, OPG, and ALP mRNAs	[158]
ICA/HA/Alginate	Freeze drying	10^−5^, 10^−6^,10^−7^ mol/l	In vitro, in vivo	The proliferation of rBMSCs was enhanced without causing harm to them. The expression levels of an osteogenic gene and the genes in the Wnt signaling pathway were increased	[139]
chitosan/gelatin/ICA multilayer-sealed TiO_2_ nanotube	Physical absorption and electrochemical anodization	0.5 mg/mL	In vitro	Osteoblastic growth was increased and the expression of bone-related genes, such as osteopontin, type I collagen, and osteoprotegerin, was enhanced. The expression of RANKL mRNA was decreased	[207]
PVA/β-TCP/ICA	Direct-ink 3D printing	0.4 g	In vitro, in vivo	Increased the adhesion and proliferation of MC-3T3-E1 cells; accelerated the in-situ bone regeneration in vivo	[156]
ICA-SH/BCP	H_2_O_2_ gas foaming	1.5 μmol	In vitro, in vivo	In vitro, the expression of angiogenic genes in human umbilical vein endothelial cells (HUVECs) was increased. This increase promoted the proliferation, migration, and osteoblastic differentiation of bone mesenchymal stem cells from ovariectomized rats (OVX-rBMSCs)	[155]
IC–CS/HA	Freeze-drying	2.0 mg	In vitro	The stimulation of alkaline phosphatase activity and mineralized nodule formation in bone marrow-derived stroma cells was promoted	[208]
CPC/ICA scaffold	Freeze-drying	10, 20, 40 µM	In vitro, in vivo	Up-regulated the expression of osteogenic and angiogenic genes in BMSCs; inhibited osteoclast; enhanced both osteogenesis and angiogenesis in the OVX calvarial defect model	[209]
ICA/micro/nano HAp granules	Wet-chemical precipitation	200, 2000 µM	In vitro, in vivo	There was an increase in ALP activity and gene expression of RUNX2, Col I, OCN, and OCN protein secretion; it also caused the expression of angiogenic genes in BMSCs such as vascular endothelial growth factor (VEGF) and angiotensin 1 (ANG1)	[210]
ICA/BioCaP granules	Co-precipitation	5, 10 mg/L	In vitro, in vivo	The critical-sized bone defects in SD rats saw a notable improvement in new bone formation	[211]
ICA/MgO/PLGA microsphere	Emulsion/solvent evaporation	0.4, 4.0, 40.0 mM	In vitro, in vivo	The expression level of RUNX2, Col I, OCN, and OCN protein secretion was increased, which effectively promoted new bone formation in rat calvarial defects	[212]
ICA/CPC@Gelatin microsphere	W/O emulsion chemically crosslinking	0.25, 0.5, 1.0, 2.0 mM	In vitro, in vivo	Promoted osteoinductivity and bone formation as well as alleviated inflammation	[213]
ICA/3MA	-	0.1, 1.0, 40 µM	In vitro, in vivo	Significantly reduced expression of senescence-associated secretory phenotype (SASP)	[197]

Accordingly, the extensive discussion on the effects of ICA in BTE reveals its promising

potential as a therapeutic agent. Multiple beneficial effects on bone tissue have been demonstrated by ICA. These effects include the promotion of bone formation, inhibition of bone absorption, activation of key signaling pathways like BMP and Wnt/β-catenin, and restoration of dysfunctional osteogenesis in senescent BMSCs. Furthermore, ICA has demonstrated the ability to alleviate inflammation in senescent macrophages, which is crucial for maintaining a healthy bone microenvironment. The studies reviewed highlight the positive impact of ICA on various aspects of bone tissue, such as enhancing osteoblast proliferation, differentiation, and mineralization, as well as reducing bone resorption and improving bone mass. Importantly, these effects have been observed without any significant side effects when ICA is used at appropriate concentrations.

Considering these findings, ICA holds great promise as a potential therapeutic agent for BTE and the treatment of bone-related conditions, including osteoporosis. Its ability to modulate critical cellular processes, activate essential signaling pathways, and support the formation of functional bone tissue makes it an attractive candidate for further research and development in the field.

## Future perspectives

The realm of employing ICA-loaded nanoplatforms for promoting osteogenesis and bone regeneration unfolds promising prospects for future research and applications. As the field advances, several directions emerge that warrant exploration and development. A paramount aspect of future research entails the refinement and optimization of nanoplatform design. Further investigation into the structural composition, size, shape, and surface properties of NFs and NPs can enhance their controlled release kinetics, stability, and interactions with bone cells. This, in turn, may lead to improved therapeutic outcomes and enhanced bioactivity. Another avenue is the exploration of multifunctional nanoplatforms. Integrating ICA with other bioactive agents or growth factors can potentially yield synergistic effects.

Such innovative combinations could extend the therapeutic capabilities of nanoplatforms, paving the way for advancements in BTE. Innovative delivery strategies represent another crucial aspect of future endeavors. Developing smart and responsive nanoplatforms that can be triggered by specific local cues within the microenvironment can enhance their therapeutic efficiency. Additionally, investigating alternative administration routes, such as minimally invasive techniques or implantable devices, could expand the scope of clinical applications.

Translating research from in vitro settings to in vivo models is paramount for validation and eventual clinical translation [214]. Rigorous preclinical studies are essential to ascertain the efficacy, safety, and long-term effects of ICA-loaded nanoplatforms. This essential step bridges the gap between laboratory findings and potential clinical trials. The trend towards personalized medicine offers a groundbreaking avenue. Tailoring ICA-loaded nanoplatforms to individual patient characteristics, including genetics, disease conditions, and bone health, holds potential for optimized therapeutic outcomes. These patient-specific approaches can mitigate adverse effects and maximize the therapeutic benefits of nanoplatform-based therapies.

Ongoing research on ICA-loaded nanoplatforms promises significant advancements in BTE and regenerative medicine. These platforms, with their combined biological and physical effects, hold the potential to revolutionize current approaches to bone regeneration. Collaboration across disciplines and sustained innovation will be the driving forces behind realizing the full potential of ICA-loaded nanoplatforms in reshaping the landscape of bone regeneration therapies.

## Conclusions

This review comprehensively investigated the diverse applications, mechanisms, and recent advancements associated with ICA-loaded nanoplatforms in promoting osteogenesis and facilitating bone regeneration. Various techniques utilized for encapsulating ICA within both nanofibers and nanoparticles are reviewed, examining their biological and physical impacts on bone tissue engineering. Numerous studies have showcased the osteogenic potential of ICA-loaded nanoplatforms and underscore their promising prospects in bone tissue engineering, despite existing challenges such as bioavailability, regulatory concerns, and commercialization hurdles.

Through a comprehensive synthesis of existing literature, valuable insights were provided into the current state-of-the-art in these field and potential avenues for future research and clinical translation introduced. The utilization of ICA-loaded nanoplatforms offers a promising strategy for effectively promoting osteogenesis and bone regeneration, warranting further investigation in this domain. As the incidence of bone disorders and fractures rises with an aging population, addressing major bone defects becomes increasingly crucial.

Icariin has shown promise in enhancing osteoblast maturation and bone formation, making it a potential treatment for osteoporosis. Its encapsulation in nanoplatforms amplifies its multifaceted effects on bone regeneration, from directing cellular differentiation to controlling inflammation and optimizing drug release. These platforms offer a versatile toolkit for fostering optimal osteogenesis, enhancing alkaline phosphatase function, raising the expression of receptor activator of nuclear factor κ-B ligand, and impacting the BMP-2/Smad4 pathway to facilitate calcified nodule formation in bone tissue. This review thus suggests that employing ICA-loaded nanoplatforms could effectively elevate the bone regeneration and osteogenesis processes.

## Data Availability

All the data that support the findings of this study are available in this manuscript.

## Abbreviations

ALP: Alkaline phosphatase
BCP: Biphasic calcium phosphate
BMSCs: Bone marrow-derived mesenchymal stromal cells
BMP-2: Bone morphogenetic protein-2
BMPs: Bone morphogenetic proteins
BTE: Bone tissue engineering
CMCS: Carboxymethyl chitosan
CAT: Catalase
CS: Chitosan
DFO: Deferoxamine
DMSO: Dimethyl sulfoxide
ECM: Extracellular matrix
EVs: Extracellular vesicles
FBS: Fetal bovine serum
GBR: Guided bone regeneration
HCC: Hepatocellular carcinoma
HPLC: High-performance liquid chromatography
HBG: Hollow bioglass
HA: Hyaluronic acid
ICA: Icariin
IL-6: Interleukin-6
LCST: Lower critical solution temperature
MSCs: Mesenchymal stromal cells
nHAP: Nano hydroxyapatite
NDs: Nanodiamonds
NFs: Nanofibers
NPs: Nanoparticles
NF-κB: Nuclear factor-kappa B
OI: Osteogenesis imperfecta
PDLA: Poly (D-lactic acid)
PNIPAAm: Poly(N-isopropylacrylamide)
PLLA: Poly(l-lactide)
PLCL: Poly(l-lactide-co-caprolactone)
PLGA: Poly(lactide-co-glycolide)
PHBV: Poly-3-hydroxybutyrate-co-3-hydroxyvalerate
PCL: Polycaprolactone
PDA: Polydopamine
PEG: Polyethylene glycol
PEO: Polyethylene oxide
PLA: Polylactic acid
PVA: Polyvinyl alcohol
RAFT: Reversible addition fragmentation chain transfer
SF: Silkdismutase
TMPZ: Tetramethylpyrazine
3D: Three-dimensional
TEM: Transmission electron microscopy
TNF-α: Tumor necrosis factor-alpha
UHPLC: Ultrahigh-performance liquid chromatography
